# Water‐Efficient Smart Drip Irrigation Enabled by a Low‐Power and High‐Efficiency Flexible Electromagnetic Actuator

**DOI:** 10.1002/advs.202514950

**Published:** 2026-01-21

**Authors:** Duo Chen, Xubin Zhu, Minwei Zhang, Kerui Li, Qinghong Zhang, Yaogang Li, Chengyi Hou, Hongzhi Wang

**Affiliations:** ^1^ State Key Laboratory of Advanced Fiber Materials College of Materials Science and Engineering Donghua University Shanghai China; ^2^ College of Smart Agriculture Xinjiang University Urumqi China; ^3^ Engineering Research Center of Advanced Glasses Manufacturing Technology Ministry of Education Donghua University Shanghai China

**Keywords:** flexible electromagnetic actuator, low‐power actuation, precision agriculture, water‐efficient irrigation

## Abstract

Global water scarcity severely restricts the sustainability of agricultural production, as conventional flood irrigation technologies exhibit low water use efficiency and insufficient flow regulation precision. Smart agriculture systems, which rely on Internet of Things (IoT) and control algorithms to establish closed‐loop irrigation frameworks, critically require intelligent fluidic actuators with rapid response, precise positioning, and broad‐range flow modulation capabilities. Here, we introduce a low‐power and high‐efficiency flexible electromagnetic actuator developed through a multi‐scale collaborative design of “magnetic materials‐flexible structures‐environmental interaction” based on an electromagnetically driven mechanism. The actuator employs low drive current and frequency modulation technology to achieve low‐power precision regulation of magnetic field strength, thereby enabling wide‐range and high‐precision flow control (0.05–5.8 mL/min). When applied to drip irrigation systems, it enables a refined, demand‐based water supply according to plants’ real‐time water requirements, with water consumption lower than that of traditional flood irrigation. This not only enhances water resource utilization efficiency but also creates an optimal moisture environment for crop growth, thereby promoting their development. It provides a viable technical pathway with economic and environmental benefits for alleviating global agricultural water scarcity pressures and advancing smart agriculture.

## Graphical abstract

Water scarcity poses a critical threat to global food security. A low‐power, flexible electromagnetic actuator is presented for smart drip irrigation systems. The device achieves precise, wide‐range flow modulation (0.05–5.8 mL/min) in response to real‐time plant demands, reducing water consumption compared to conventional flood irrigation. This technology advances water‐use efficiency and crop productivity, offering a scalable solution for sustainable precision agriculture.

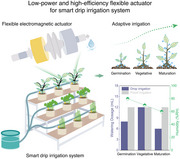

## 1 Introduction

Global water scarcity is increasingly severe, and agricultural irrigation, as a primary water‐consuming sector, urgently requires technological innovation to achieve efficient water use and sustainable development [1, 2]. Conventional irrigation methods, such as flood irrigation, suffer from serious water waste and low efficiency. Statistics show that water loss rates caused by evaporation and seepage during flood irrigation exceed 40% [3, 4, 5]. Drip irrigation systems are a core method for precision irrigation and efficient water conservation, with their precision, reliability, and environmental adaptability directly determining water use efficiency [6, 7]. These systems deliver water and nutrients directly to crops through pipeline networks, featuring low flow rates and high‐frequency delivery to significantly reduce evaporation and deep percolation [8, 9]. However, existing drip irrigation systems have engineering designs lacking precise models, leading to poor matching between pipe network layouts and crop water demand characteristics [10], making it difficult to adapt to refined requirements. Moreover, current drip irrigation actuators have issues such as low flow control precision and obvious response lag [11, 12]. The stability and repeatability of micro‐flow regulation struggle to meet the requirements of precision irrigation [13, 14], while their coordination with Internet of Things (IoT) sensors and intelligent control systems needs improvement. These technical bottlenecks significantly restrict the intelligent and efficient development of drip irrigation systems [15]. In this context, developing new actuators with flexible deformation, low‐power driving, and high environmental tolerance has become an urgent need for smart agriculture technology upgrades [16].

In contemporary drip irrigation systems, actuator materials primarily comprise engineering plastics and metal alloys [17, 18]. Engineering plastics, favored for their corrosion resistance and cost‐effectiveness [19, 20], exhibit limited mechanical durability under prolonged environmental stress. Metal alloys, despite their robustness in high‐pressure scenarios [21, 22], are susceptible to chemical degradation and economic infeasibility. Conversely, flexible actuators leveraging elastic polymers and composites demonstrate superior adaptability and dynamic responsiveness [23, 24], with structural plasticity mitigating environmental interference [25]. Modular integration of sensing and actuation units further enhances system synergy [26, 27, 28]. However, material longevity and scalable manufacturing remain unresolved challenges. Wang et al. [29] introduced a polydimethylsiloxane (PDMS)‐based dielectric elastomer pump achieving a 0.35 mL/min flow rate, yet its high‐voltage requirement restricts practicality in low‐energy agricultural settings [30, 31]. Zhao et al. [32] proposed a magnetically controlled PDMS tubular actuator (MTLA‐SIS) utilizing Laplace pressure gradients for droplet manipulation, though challenges persist in field deployment, including magnetic infrastructure costs, pore clogging from agrochemical residues [33], and maintenance complexities [34].

To address challenges of high water loss in flood irrigation and deficiencies in current drip irrigation systems, including inadequate precision control, low‐accuracy micro‐flow regulation, response hysteresis, and poor environmental adaptability, we propose a flexible electromagnetic actuator based on an electromagnetically driven mechanism. Using multi‐scale co‐design methodology, we developed a system integrating a corrugated silicone matrix doped with magnetic particles (4 mm pitch, 200 µm wall thickness), permanent magnets, excitation coils, and a closed‐loop control system. This configuration leverages low‐coercivity magnetic materials to enable rapid magneto‐responsive actuation, overcoming the bottleneck of achieving wide‐range flow regulation (0.05–5.8 mL/min) with high precision under low driving currents. The incorporated “actuation‐response‐holding” full‐cycle control logic provides an efficient solution for smart agricultural micro‐irrigation systems. Moreover, it establishes a generalized magneto‐electrical theoretical framework for multi‐physics‐coupled design of magnetic soft robots in harsh environments, advancing water‐efficient irrigation toward intelligent adaptivity while demonstrating significant technical and socioeconomic value.

## 2 Results

### 2.1 Flexible Electromagnetic Actuator

**FIGURE 1:**
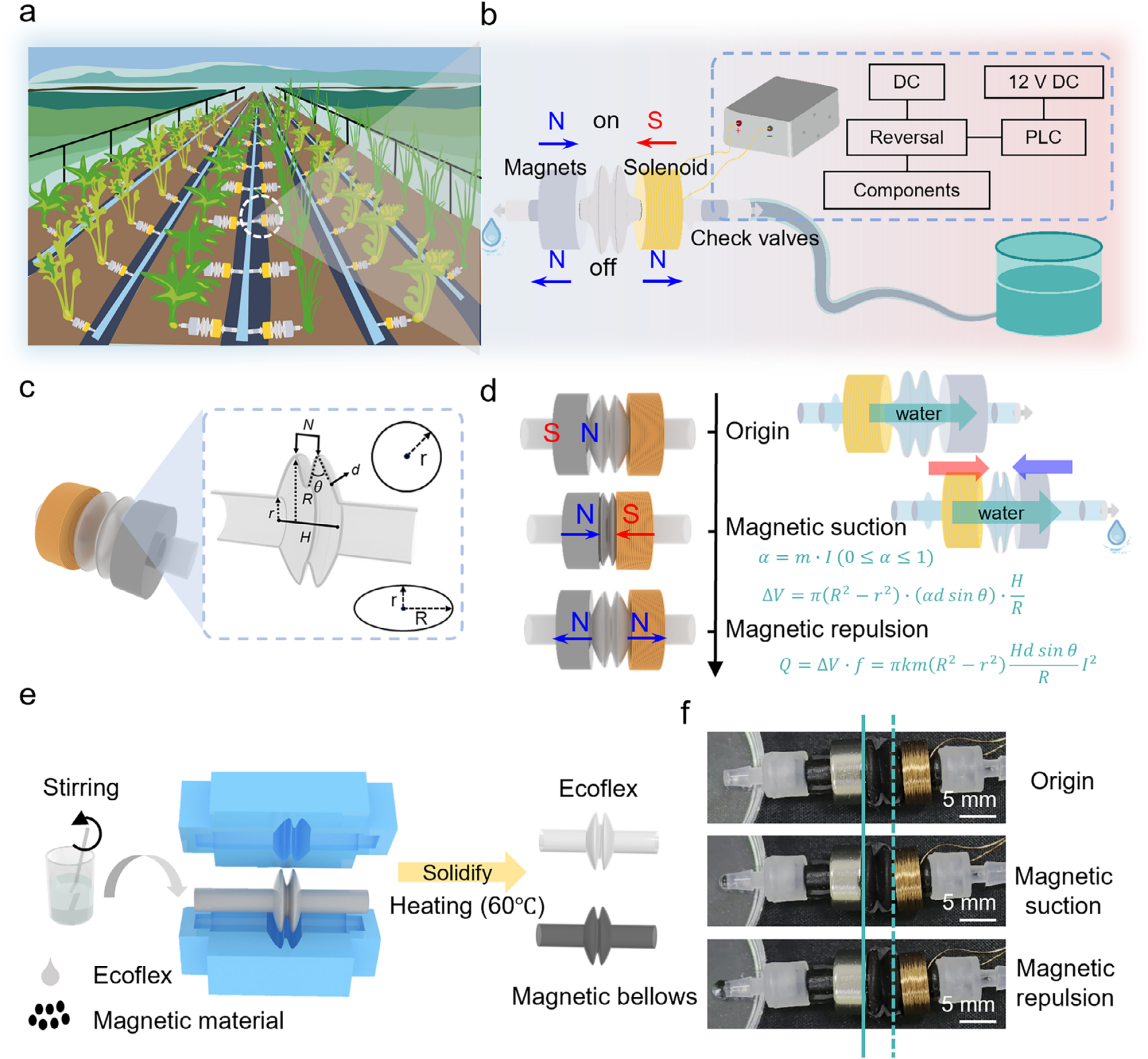
Flexible electromagnetic actuator. (a) Schematic of parallel‐integrated smart agriculture precision irrigation system with independent control. (b) The actuator components include a permanent magnet, a corrugated matrix of silicone, induction coils, and unidirectional dropwise transport facilitated by the application of an electrical current and a check valve. The dimensions of the corrugated silicone matrix are as follows: corrugation pitch N of 4 millimeters, wall thickness of 200 micrometers, interior radius r of 2 millimeters, and (c) rest radius R of 5 millimeters. (d) Electromagnetically driven periodic deformation mechanism under field‐stress–strain coupling. (e) Fabrication workflow for pure Ecoflex and magnetic particle‐doped corrugated matrices. (f) The actuator pumping mechanism involves the induction of heteropolar coupling for axial compression (fluid extrusion) by means of a forward current. In the reverse current scenario, homopolar repulsion enables elastic reset and pressure gradient‐driven directional transport.

Efficient water utilization remains a critical challenge in smart agriculture [35, 36]. Conventional irrigation methods fail to meet modern agricultural demands for precise water management, often causing localized drought or excessive water waste [37, 38]. The proposed flexible electromagnetic actuator addresses this via parallel circuit integration, enabling real‐time adjustment of water flow rate and irrigation duration based on individual plant water demand. This forms an independently controllable, parallel‐integrated smart agricultural system (Figure 1a). Comprising a permanent magnet, corrugated matrix, and induction coils (Figure 1b), the actuator achieves precise structural modulation through electromagnetic control of magnetic field intensity. Magnetic coupling between the induction coil and magnet drives the squeezing deformation of the flexible matrix, with check valves ensuring unidirectional fluid transport. The check valves in this system function through a two‐phase cycle: In the compression phase, driven by electromagnetic force, the chamber pressure rises, inducing closure of the inlet valve and opening of the outlet valve to pump fluid forward. During the subsequent elastic reset phase, negative pressure is established inside the chamber. Herein, the outlet valve seals to prevent fluid backflow, while the inlet valve opens to draw fluid from the water source in preparation for the next operational cycle. This mechanism inherently ensures net unidirectional fluid transport under periodic actuation. The core structure of the actuator adopts a corrugated substrate design (Figure 1c), comprising two corrugated structures. In this structure, *R* denotes the radius of the corrugation, *r* denotes the pipe radius, 𝜃 denotes the deformation angle of the corrugation, *d* denotes the deformation depth, and *N* denotes the width between two crests. The degree of corrugation bending is correlated with the corrugation radius and width. Through optimization of the central angle 𝜃 of the corrugation, magnetic field pressure output, and structural dimensions, the performance of the actuator can be effectively controlled to better adapt to requirements under different operating conditions and enhance its reliability and efficiency in various applications.

The actuation system relies on synergistic interaction between the corrugated elastic matrix and permanent electromagnetic coil assembly (Figure 1d). Under conditions of forward current excitation, heteropolar magnetic attraction induces axial compression of corrugations; conversely, reverse current triggers homopolar repulsion for elastic reset, thereby creating periodic “compression‐reset” actuation cycles (Figure S1). The compression coefficient α (0≤ α ≤1) is defined by (Note S1):

(1) $$\alpha =m\cdot I\left(0\leq \alpha \leq 1\right)$$

where m is the material constant and I is the driving current. The corresponding flow volume ΔV follows:

(2) $$\Delta V=\pi \left(R^{2}-r^{2}\right)\cdot \left(\alpha d\sin\theta \right)\cdot \frac{H}{R}$$

At full compression, flow rate Q is expressed as [39, 40]:

(3) $$Q\;=\Delta V\cdot f\;=\pi \mathrm{km}\left(R^{2}-r^{2}\right)\frac{\mathrm{Hd}\sin\theta }{R}I^{2}$$

where f is the frequency coefficient. Fabrication of the corrugated elastic matrix involves injecting Ecoflex silicone prepolymer into custom molds, followed by thermal curing at 60°C for 1 h to induce cross‐linking. Magnetic responsiveness is achieved by dispersing magnetic particles into the silicone precursor via mechanical stirring and vacuum degassing, forming magnetically active polymer composites (Figure 1e). Under a 50 mA current, forward excitation drives axial compression for fluid extrusion, while reverse excitation enables elastic reset for droplet accumulation (Figure 1f). This cyclic polarity switching realizes controlled, dropwise fluid transport. Through electromagnetic control strategies and flexible material design, the proposed actuator delivers tunable performance, offering a robust solution for smart agricultural irrigation systems.

### 2.2 Characterization of Corrugated Matrix

**FIGURE 2:**
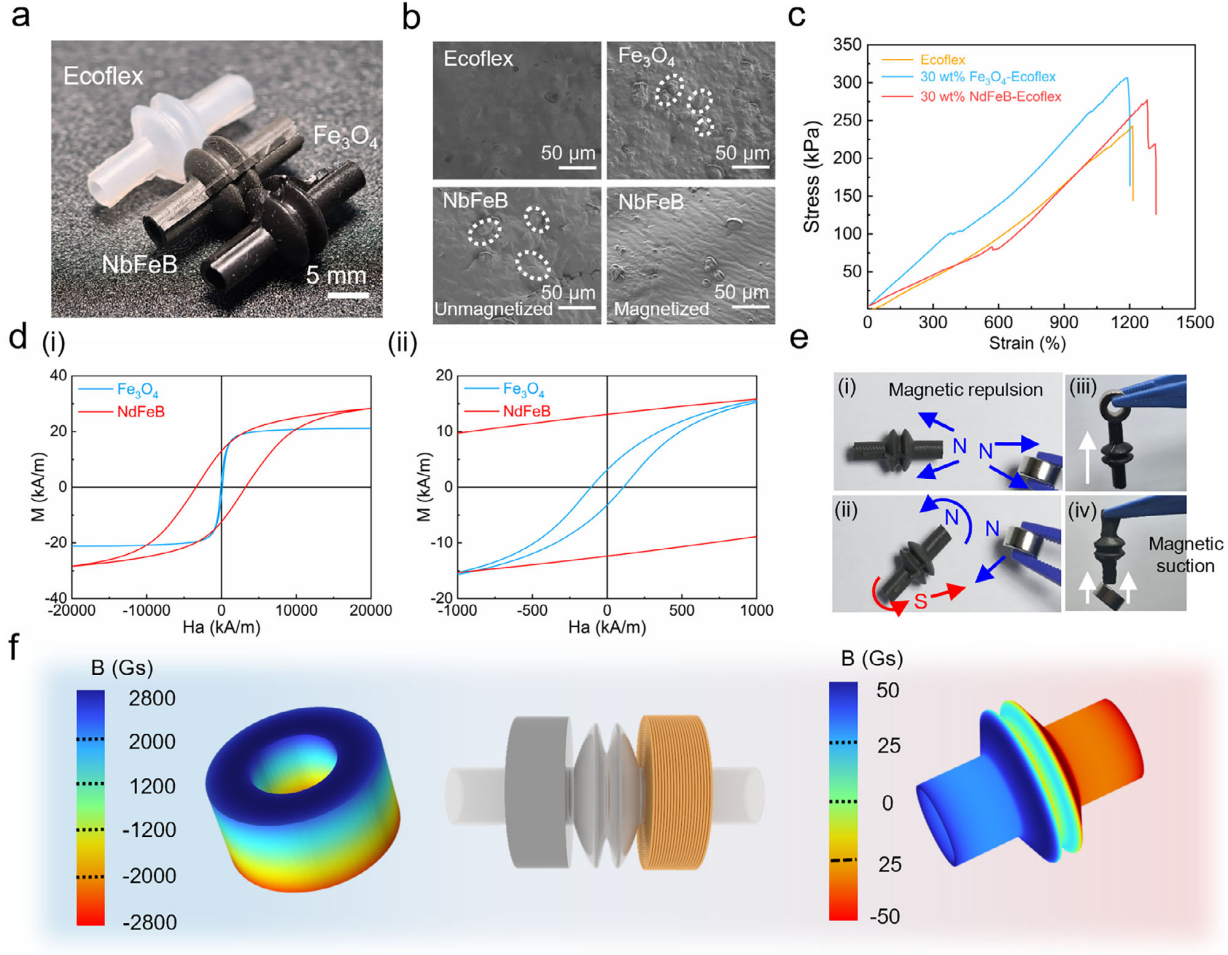
Characterization of corrugated matrix. (a) Macroscopic morphology comparison of Ecoflex 00–30, Fe_3_O_4_, and NdFeB materials. (b) Scanning electron microscopy (SEM) images of silicone matrix (amorphous structure), Fe_3_O_4_ nanoparticle‐doped matrix (20 µm particle size), and magnetized/unmagnetized NdFeB‐doped matrix (5 µm particle size). (c) Tensile property comparison of Ecoflex 00–30 Fe_3_O_4_ and NdFeB materials. (d) Hysteresis loops of Fe_3_O_4_ and NdFeB, including (i) static hysteresis loops measured by a vibrating sample magnetometer (VSM), and (ii) magnified central portions of loops highlighting remanence and coercivity. (e) Dynamic response demonstration of NdFeB composite corrugated matrix under magnetic field, showing distinct attraction and repulsion effects (i, ii), and of Fe_3_O_4_ composite corrugated matrix attracting a 10 g magnet (iii, iv). (f) Magnetic flux density distribution between the NdFeB permanent magnet (grade N52 dimensions; inner diameter 4 mm, outer diameter 10 mm, thickness 4 mm) and the NdFeB composite in the actuation unit.

The flexible electromagnetic actuator's core driving force for efficient fluid pumping originates from its corrugated magnetic silicone substrate, combining superior mechanical and magnetic properties. Fabricated via a casting process with Ecoflex 00–30 silicone in custom molds, the substrate exhibits a metallic luster with 30 wt.% NdFeB magnetic powder doping (Figure S2) and a dark gray appearance with 30 wt.% Fe_3_O_4_ doping (Figure 2a). This 30 wt.% loading optimizes magnetic enhancement and structural integrity, as lower levels fail to boost magnetic fields sufficiently, while higher levels introduce matrix defects. SEM analysis shows pure silicone cross‐sections (Figure 2b) have smooth surfaces, whereas Fe_3_O_4_‐silicone cross‐sections (Figure S3) reveal uniformly embedded bright‐region particles in the dark matrix with good encapsulation, despite occasional local agglomeration. NdFeB‐silicone cross‐sections show 5 µm particles fully encapsulated without defects when unmagnetized (lower‐left), but show partial protrusion from the silicone coating upon magnetization (lower‐right), indicating weak matrix suppression. Local protrusions observed on the surface of silicone matrices embedded with magnetized NdFeB particles arise from the strong intrinsic magnetic moments formed in high‐remanence NdFeB particles after magnetization. The interparticle magnetic dipole‐dipole repulsive forces generated thereby may partially surpass the mechanical binding capacity of the silicone matrix. This microstructural heterogeneity modulates the dynamic mechanical properties via two synergistic effects. First, in the periodic deformation process driven by an alternating magnetic field, these protruding hard‐phase particles become local stress concentration hotspots, facilitating the initiation and propagation of fatigue cracks in the matrix. Second, relative sliding at the particle‐matrix interface induces supplementary internal frictional dissipation, leading to a reduction in the energy conversion efficiency from magnetic energy to mechanical work [41]. Uniform particle distribution is critical for actuator performance consistency, durability, and stress transfer during “compression‐return” cycles (Figure 2c). Hysteresis loop tests (Figure 2d) confirm NdFeB's high saturation magnetization, remanence, and coercivity (hard magnetic) [42], while Fe_3_O_4_ exhibits soft magnetic behavior with near‐zero remanence and low coercivity [43], enabling fast magnetic response, millisecond‐scale deformation, and low hysteresis loss for high‐frequency control. Demonstrations (Figure 2e) show magnetized NdFeB attracts/repels magnets (Figure S4), while Fe_3_O_4_‐doped samples lift a 10 g magnet (Figure S5). Hall sensor measurements (Figure 2f) reveal the magnet's 2800 Gs field strength and the substrate's 50 Gs contribution, synergizing to enable precise deformation control and stable fluid pumping. This design endows the actuator with high efficiency, low power consumption, and reliability for smart agricultural applications.

### 2.3 Characterization of Flexible Electromagnetic Actuator

**FIGURE 3:**
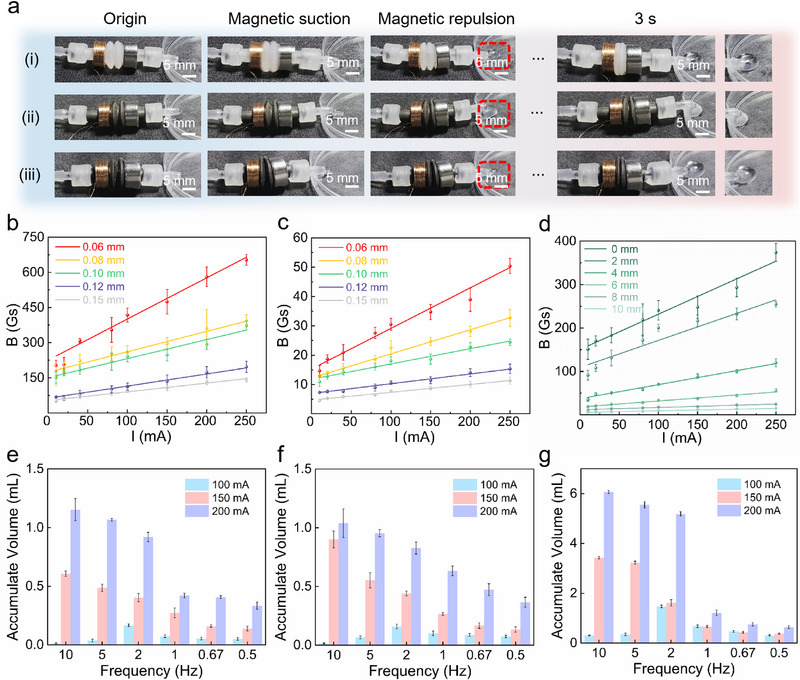
Characterization of flexible electromagnetic actuator. (a) The dynamic behavior of fluid actuation of Ecoflex matrix, Fe_3_O_4_, and NdFeB composite corrugated matrices under current excitation (50 mA) was recorded using high‐speed photography. (b) Surface magnetic flux density distributions of coils with different wire diameters (0.06, 0.08, 0.10, 0.12, and 0.15 mm). (c) radial corrugation distance versus magnetic induction intensity under varying currents (50–250 mA). (d) Axial magnetic induction decay curve within a radial corrugation range (0–10 mm) for a 0.08 mm wire diameter coil. (e) Flow rate comparisons of three actuator types under frequency‐modulated actuation (0–10 Hz): Ecoflex matrix exhibits non‐monotonic flow‐frequency response due to viscoelastic relaxation at low current (100 mA), with peak flow Q_max_ of 1.2 mL/min at 2 Hz; linear flow increase with frequency occurs when magnetostrictive stress exceeds the viscoelastic threshold at higher currents. (f) NdFeB composites show limited magneto‐mechanical conversion efficiency due to magnetic domain pinning from hard magnetic properties, with a maximum flow rate of 1.0 mL/min across all frequencies (83.3% lower than Fe_3_O_4_ systems). (g) Fe_3_O_4_ composites achieve linear flow‐frequency response (Q_max_ 5.8 mL/min at 200 mA and 10 Hz) via soft magnetic properties, with unit frequency flow gain ΔQ/Δf of 0.58 mL/(min·Hz), which is four times that of the Ecoflex matrix, highlighting advantages in wide‐frequency (0.1–10 Hz) pumping scenarios.

Dynamic pumping processes of three materials under 100 mA low‐current excitation were captured via high‐speed photography (Figure 3a). Driven by alternating magnetic fields, corrugated silicone matrices of Ecoflex (Figure 3a(i)), Fe_3_O_4_‐silicone composite (Figure 3a(ii)), and NdFeB‐silicone composite (Figure 3a(iii)) all exhibited uniform axial compression to drive fluid transport (Movie S1). Ecoflex showed a flexible response at low current, achieving stable pumping via periodic deformation. Fe_3_O_4_‐silicone composites utilized magnetic enhancement for efficient fluid transport through axial deformation under low current; however, NdFeB‐silicone composites, due to high remanence, rapidly extruded fluid in forward magnetic fields but suffered flow decline from magnetic attraction during reverse fields. This discrepancy highlights the critical role of corrugation design and precise magnetic field direction control in maintaining pumping efficiency.

Induction coil wire diameter is a key factor affecting magnetic field distribution and actuation efficiency. A systematic study was performed in which the surface magnetic induction and field distribution at varying distances were investigated for wire diameters of 0.06, 0.08, 0.10, 0.12, and 0.15 mm. The fixed coil geometry used in this study was 4 mm in length, 10 mm in outer diameter, and 4 mm in thickness (Figure 3b,c). A coil with a diameter of 0.08 mm and an internal electric current of 200 mA demonstrated optimal magnetic properties, characterized by a surface magnetic induction of 400 Gs and a radial magnetic intensity of 30 Gs along the corrugation length. This coil exhibited a 10% increase in magnetic intensity compared to coils with a diameter of 0.10 mm, and a 50% increase compared to coils with a diameter of 0.12 mm. This ensures sufficient actuation (>20 Gs threshold) while optimally regulating current conduction to suppress Joule heating [44].

The magnetic field distributions of induction coils were measured using Hall sensors (Figure 3d). The data demonstrated a decline in magnetic intensity with radial distance, yet a persistence of >30 Gs within the deformation range (r <10 mm). This finding aligns with the magnetization characteristics of magnetic composites, which are designed to optimize actuation efficiency. The pumping flow dynamics of three systems (Ecoflex matrix (Figure 3e), NdFeB‐doped composite (Figure 3f), and Fe_3_O_4_‐doped composite (Figure 3g) were tested across a frequency range of 0–10 Hz under varying currents (0‐200 mA). Under a low current of 100 mA, all three actuator configurations displayed non‐linear flow‐frequency behaviors. For the Ecoflex‐based matrix, a peak flow rate of 1.2 mL/min was attained at 2 Hz; flow rates declined at higher frequencies owing to phase mismatch arising from viscoelastic lag [45]. Elevating the current mitigated this phase mismatch, resulting in a positive flow‐frequency correlation across the 2–10 Hz range. This phenomenon is a quintessential reflection of the coupling effect between material viscoelasticity and external periodic driving loads. The peak frequency corresponds to an approximate resonant matching condition between the driving period and the material's characteristic relaxation time scale, which is associated with the specific stress magnitude under the applied driving parameters. At this resonant frequency, the silicone network possesses sufficient time to accomplish most of its elastic deformation within one half‐cycle, while viscous resistance does not yet fully govern the dynamic behavior [46] (Note S1). When the driving frequency substantially surpasses this characteristic value, the material's viscous response lag is exacerbated, causing the deformation amplitude to be unable to track the rapid magnetic field switching. This compromises the effective pumping stroke, ultimately leading to a reduced flow rate. The Fe_3_O_4_ composites demonstrated soft magnetic properties, facilitating rapid magnetic moment reorientation under weak fields. This process resulted in efficient periodic wall deformation through magnetostriction, generating a flow rate of 5.8 mL/min at 200 mA. This is 5–6× higher than the levels observed in Ecoflex and NdFeB. The composites also exhibited a high‐frequency response, rendering them suitable for dynamic irrigation applications. NdFeB composites, hindered by magnetic domain pinning from their inherent hard magnetic properties [47], demonstrated low energy conversion at low fields, necessitating gradient fields for steady‐state magnetization locking. This material‐specific functional differentiation provides flexible solutions for multi‐mode regulation in smart agricultural irrigation systems and offers design insights for optimizing magnetic composites in a flexible electromagnetic actuator.

### 2.4 Demonstration of Smart Drip Irrigation Based on Electromagnetically Driven Flexible Actuators

**FIGURE 4:**
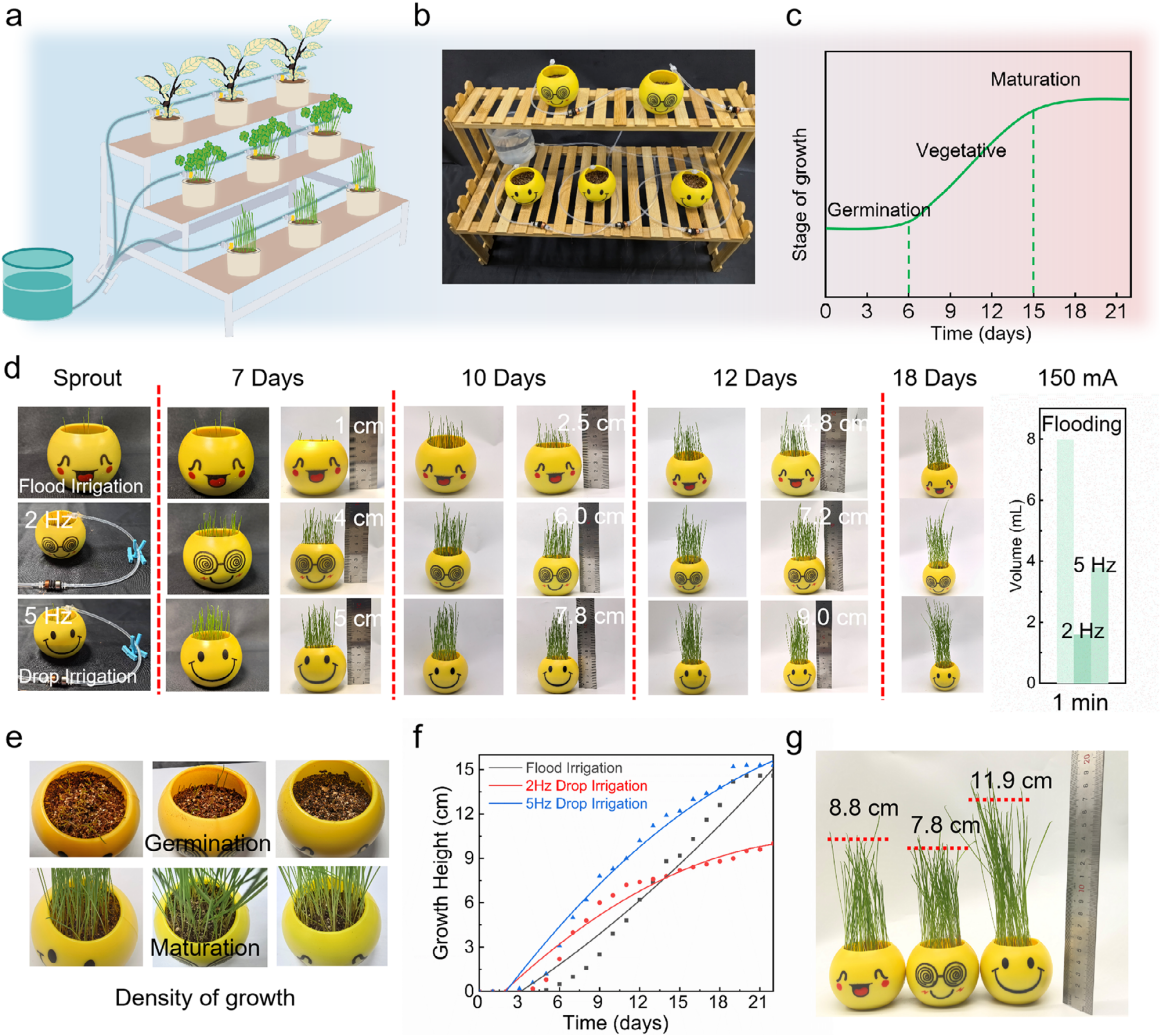
Demonstration of smart drip irrigation based on electromagnetically driven flexible actuators. (a) Schematic diagram of the electromagnetically driven drip irrigation system architecture. (b) Laboratory performance test of dropwise irrigation, demonstrated under controlled environmental conditions (25 ± 1°C/60%RH). (c) Plant growth cycle observation timeline. (d) Experimental design of irrigation mode dependence on plant water uptake: I. conventional flood irrigation; II. low‐frequency irrigation (2 Hz); III. high‐frequency irrigation (5 Hz). (e) Plant vigor comparison after one week of irrigation. (f) The three‐week dynamic growth trajectory curves of the plants demonstrate that the 5 Hz drip irrigation group achieved the maximum growth rate (µ_max_ of 2.1 cm/d), representing a 61.5% increase compared to the flood irrigation group (µ_max_ of 1.3 cm/d) and a 40% reduction in lag phase. However, the flood irrigation group eventually caught up with the 2 Hz drip group in growth rate. g Plant height comparison among three irrigation modes after three weeks.

The drip irrigation system consists of the flexible electromagnetic actuator unit and the fluidic channel (Figure 4a). Cultivation was conducted under controlled laboratory conditions (25°C ± 1°C, 60% ± 5%RH) (Figure 4b), using standardized plastic pots (base diameter: 6.5 cm, height: 5.2 cm) aligned with the growth cycle of wheatgrass [48] (Figure 4c; Note S2). A comparative irrigation design was implemented, including flood irrigation (8 mL daily) and drip irrigation at 2 Hz (1.6 mL/min) and 5 Hz (3.6 mL/min) (Figure S6; Movie S2). Growth dynamics were measured at 7, 10, 12, and 18 days post‐planting. The plants that received flood irrigation exhibited heights of 1, 2.5, 4.8, and 10.2 centimeters, while those subjected to 2 Hz drip irrigation demonstrated heights of 4, 6, 7.2, and 9.6 centimeters, respectively. The 5 Hz drip irrigation group exhibited superior performance, with heights of 5, 7.8, 9, and 12.8 centimeters (Figure 4d). Visual observations from germination to maturity confirmed robust growth under both drip and flood irrigation (Figure 4e; Figure S7). Flood irrigation induced accelerated vertical growth in the latter stages, achieving approximately 15 centimeters at day 21. Conversely, 5 Hz drip irrigation exhibited faster early growth and reached 15 centimeters at maturity. The 2 Hz drip irrigation group exhibited a more gradual and consistent growth trajectory, attaining a length of 9 centimeters after a period of three weeks. Collectively, flood irrigation and 5 Hz drip irrigation were most effective for height development (Figure 4f).

At two weeks post‐maturity (day 21), the 5 Hz drip irrigation group achieved significantly higher plant heights (11.9 ± 1.6 cm) compared to flood irrigation (8.8 ± 1.1 cm) and 2 Hz drip irrigation (7.8 ± 0.6 cm) (Figure 4g; Figure S8). These results highlight the water‐saving and growth‐promoting advantages of high‐frequency drip irrigation, particularly during early developmental stages, while flood irrigation remains effective for late‐stage biomass accumulation. Drip irrigation using a flexible electromagnetic actuator and fluidic channels offers a combination of low power consumption and high efficiency. At 2 Hz, drip irrigation substantially increases plant growth while significantly reducing water usage. At 5 Hz, the growth rate and maturity height of plants increase, and drip irrigation outperforms flood irrigation in terms of water efficiency. The innovative solution for smart water‐saving agriculture is an example of the synergy between low‐power electromagnetic actuation and high‐efficiency irrigation, which delivers water‐saving benefits. Versus commercially available systems, the flexible electromagnetic actuator fundamentally exhibits inherent advantages in low‐power actuation and milliliter‐scale flow precision control, aligning with the demand for miniaturized, distributed, and precisely controllable actuation units in smart agriculture [49] (Table S1). The 5 Hz smart drip irrigation system used only 73.4% of the total water consumed by flood irrigation over 22 days (representing a 26.6% water saving rate), while simultaneously achieving a significantly higher average plant height of 11.9 ± 1.6 cm (an increase of approximately 35% compared to 8.8 ± 1.1 cm for flood irrigation). This achievement synergistically realizes water conservation and crop growth promotion, with promising prospects for diverse plants’ drip irrigation and an efficient, precise irrigation solution for smart agriculture (Table S2).

### 2.5 Growth Regulation of Drip Irrigation and Analysis of Water Saving in Xinjiang, China

**FIGURE 5:**
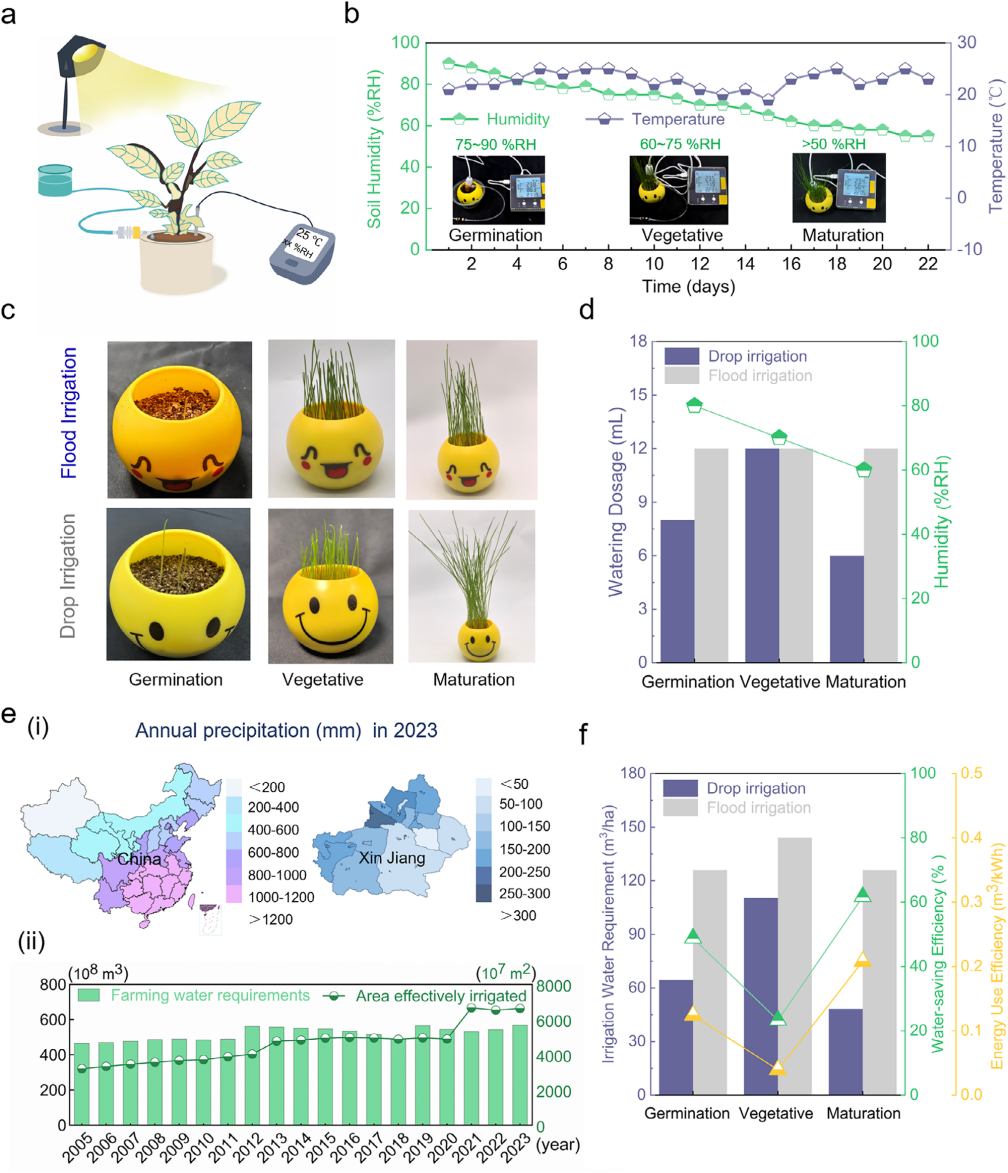
Growth regulation of drip irrigation and analysis of water saving in Xinjiang, China. (a) Plant cultivation schematic. (b) Temperature and humidity control curves during plant growth cycles. (c) Growth comparisons between flood irrigation and dropwise irrigation at Germination, Vegetative, and Maturation stages. (d) Water usage comparison between flood irrigation and 5 Hz drip irrigation throughout the experimental period. (e) Annual precipitation in China and the Xinjiang region in 2023 (i), and trends in agricultural water consumption and effective irrigated area in the Xinjiang region from 2005 to 2023 (ii). (f) Calculation and analysis of water saved, water saving rate, and energy consumption efficiency for 1 hectare of farmland in Xinjiang based on plant growth cycles.

For further comparison between 5 Hz drip irrigation and flood irrigation, during cultivation, soil moisture, temperature, and supplementary lighting were controlled to establish an optimal growing environment [50] (Figure 5a). Throughout the growth stages, watering regimes were adjusted according to plant physiology and soil moisture dynamics, with humidity maintained at 75%–90% RH during germination, 60%–75% RH during vegetative growth, and >50% RH during maturity (Movie S3). The temperature‐humidity control curves over the experimental cycle (Figure 5b) provided a stable microclimate for plant development,[51] (Figure S9),. Based on pot volume, flood irrigation delivered a constant 8 mL of water daily, while 5 Hz drip irrigation (3.6 mL/min) adjusted water input by growth stage: 8 mL/day for the 7‐day germination phase, 12 mL/day for the 8‐day vegetative stage, and 6 mL/day for the 7‐day maturity period. Plants exhibited healthy growth across all stages (Figure 5c), with drip irrigation requiring only 73.4% of flood irrigation's total water volume to achieve superior growth performance (Figure 5d).

In 2023, China's average annual precipitation was recorded at 642.8 mm [52], while the Xinjiang region registered a significantly lower value of 154.0 mm [53], representing less than 23% of the national mean (Figure 5e(i)). This disparity accentuates the region's pronounced aridity and the imbalanced spatiotemporal precipitation patterns (Figure S10). Time‐series data for agricultural water use and effective irrigation area in Xinjiang (2005–2023) [53] show total water consumption increased from 46.4 to 56.8 billion m^3^, while effective irrigation area expanded from 32.04 to 66.29 million hectares. Concurrently, water use per hectare decreased by 40.83% (from 1448.19 to 856.84 m^3^/hm^2^) (Figure 5e(ii)), underscoring the critical role of drip irrigation technologies (Figure S11).

Based on laboratory planting data, the quantitative assessment of a 1‐hectare farmland in Xinjiang was conducted with data correction calculations performed in accordance with the flood irrigation and drip irrigation loss rate models specific to the region [54, 55]. Considering the differences in irrigation efficiency under Xinjiang's arid ecological conditions, the field water use coefficients for drip irrigation and flood irrigation were set at 0.90 and 0.50, respectively (corresponding to irrigation loss rates of 15% and 50%). Targeting the high‐temperature and sandy wind environment, an environmental compensation coefficient of 1.25 and an intelligent regulation coefficient of 0.85 were introduced, with the comprehensive energy consumption correction factor being 1.0625 (Note S3). The quantitative assessment of a 1‐hectare farm in Xinjiang, adjusted for flood/drip irrigation loss rates, reveals that traditional flood irrigation requires 396 m^3^ of water over a 22‐day cycle. In contrast, the smart drip system, which is controlled by electromagnetically driven flexible actuators, reduces water consumption to 223.1 m^3^ (43.7% water saving), with a comprehensive energy efficiency of 0.1004 m^3^/kWh (Figure 5f). This “high‐frequency micro‐irrigation” model significantly increases annual water savings in Xinjiang's pilot zones (Tables S3 and S4). When paired with a photovoltaic‐magnetic energy storage combined power supply system [56], it achieves a reduction in the full‐life‐cycle carbon footprint. Electromagnetically controlled drip irrigation, through “material‐water‐energy” tripartite innovation, provides a paradigm‐level solution to the optimization of the “water‐energy‐food” triangle in arid‐region agriculture [57, 58].

## 3 Discussion

In conclusion, we have developed a novel flexible electromagnetic actuator. This actuator employs a synergistic “corrugated substrate‐electromagnetic coupling” architecture, enabling precise dropwise fluid actuation. This design facilitates broad‐range flow regulation under low‐current conditions while preserving exceptional responsiveness to high‐frequency pulse signals. When integrated with plant irrigation systems, the actuator offers significant benefits in the realm of smart agriculture. A 5 Hz high‐frequency pulsation drip mode has been shown to enhance the efficiency of root development during the germination phase. Additionally, dynamic soil moisture regulations have been observed to reduce water consumption per unit yield. This suggests the potential for annual water savings of 5832 m^3^ per hectare in arid regions such as Xinjiang. When employed in conjunction with photovoltaic‐storage systems, it leads to a further reduction in agricultural carbon footprints. The technology extends to precision nutrient solution delivery in controlled‐environment agriculture and intelligent water cycling for desertification control, providing material‐driven technical support for sustainable smart agriculture paradigms that synergistically optimize the “water‐energy‐food” system.

## 4 Methods

### 4.1 Fabrication of Corrugated Matrix Molds via 3D Printing

A hollow mold with corrugated structures was designed using SOLIDWORKS. The design consists of two external support molds and an internal hollow support mold. The external mold dimensions are as follows: The internal mold dimensions were adjusted according to the design specifications, resulting in measurements of 49.5 millimeters in length, 15.0 millimeters in width, and 7.0 millimeters in thickness. The molds were fabricated using an stereolithography (SLA) 3D printer (model from Lingxi Zhizao Tianjin Technology Co., Ltd., China), with Crysta‐7 material serving as the printing substrate. Subsequent to the fabrication process, the molds were meticulously cleaned using an ultrasonic cleaner, rinsed, and dried. A release agent (Ease Release 200, Smooth‐On, USA) was applied to the inner surfaces of the molds to prevent adhesion of silicone and its composites, thereby facilitating smooth demolding during the preparation of corrugated matrices. This process ensures precise replication of corrugated geometries and enables consistent production of flexible magnetic‐responsive matrices for subsequent fluidic actuation applications.

### 4.2 Preparation of Corrugated Matrices with Different Materials

For the pure Ecoflex silicone corrugated matrix, Ecoflex00‐30 silicone prepolymer (Smooth‐On, USA) was weighed at a mass ratio of A:B = 1:1 (5 g of each component). To prepare the 30 wt.% Fe_3_O_4_‐doped magnetic silicone corrugated matrix, 3 g of Fe_3_O_4_ particles (100 µm, Shanghai Titan Co., Ltd.) were mixed with 7 g of silicone (3.5 g of component A + 3.5 g of component B). For the 30 wt.% NdFeB‐doped magnetic silicone corrugated matrix, 3 g of NdFeB particles (5 µm, Guangzhou Xinnuode Transmission Components Co., Ltd.) were combined with 7 g of silicone (3.5 g of component A + 3.5 g of component B). Fe_3_O_4_ and NdFeB powders were dried in a vacuum oven at 60°C under −0.1 MPa for 2 h to remove moisture. The dried particles were then dispersed in anhydrous ethanol via ultrasonic treatment (100 W, 10 min) to enhance compatibility with the silicone. The particles were added to component A in batches, followed by magnetic stirring at 500 rpm for 30 min and ultrasonic treatment for 15 min. After adding component B, the mixture was re‐stirred at 300 rpm for 10 min until homogeneous. The mixture was transferred to a vacuum degasser (−0.095 MPa) for 15 min to eliminate bubbles. The inner walls of the molds were evenly coated with release agent and preheated to 40°C. The mixture was slowly injected into the molds, followed by secondary vacuum degassing for 5 min. The molds were cured in a constant‐temperature oven at 60°C for 2 h. After curing, the corrugated matrices were separated by gentle tapping, with edge burrs trimmed and the matrices left to stand for 24 h to ensure complete curing. NdFeB‐doped composite corrugated matrices were magnetized after fabrication.

### 4.3 Characterization of Flexible Electromagnetic Actuators

To characterize the magnetic Fe_3_O_4_ and NdFeB corrugated matrix actuators based on iron oxide/neodymium‐iron–boron concentration and distribution, particle dispersion, mechanical properties, hysteresis loops, and magnetic field strength were systematically analyzed to evaluate matrix characteristics. For particle distribution observation, a field‐emission scanning electron microscope (Zeiss Sigma 360 with Oxford Xplore 30 EDS) was used. Mechanical properties of corrugated matrices with different materials were assessed via tensile testing using an electronic universal material testing machine (Instron 5969, USA). Magnetic hysteresis loops and magnetic induction intensity of composite corrugated matrices were measured using a vibrating sample magnetometer (VSM, Lake Shore, USA) and a gaussmeter (DX‐102F), with signal acquisition for data plotting. For droplet‐based outflow quantification, water was collected in discrete sampling at 10‐s intervals over 1 min. The flow rate was determined as the time‐averaged value from 3 independent measurements per actuation condition (e.g., 200 mA, 10 Hz), ensuring statistical robustness against transient fluctuations.

### 4.4 Actuator Assembly and Circuit Setup

A precision programmable power supply (3A, 60V, Qiuyin Electronics Shanghai Co., Ltd.) was used to deliver direct current (DC) current. The direction of the current was controlled via an isolated DC motor forward‐reverse module (Hangzhou Longke Electronics Co., Ltd.), and automated control was achieved using a programmable logic controller (PLC) all‐in‐one controller (Aoshangming Technology Co., Ltd.). Both ends of the corrugated matrix were inserted into a hollow magnet and an induction coil, respectively, to ensure a tight fit. Wires led out from both ends of the coil were connected to the output terminals of the forward‐reverse module, while the output of the precision programmable power supply was linked to the input terminals of the forward‐reverse module, completing the actuation circuit. This configuration enables precise control over current direction and intensity for magnetic actuation.

### 4.5 Drip Irrigation Demonstration with Wheatgrass

Drip irrigation technology was demonstrated using wheatgrass as the experimental model to observe dynamic changes in plant height across different growth stages under drip irrigation. Experiments were conducted in a laboratory with environmental parameters strictly controlled at 25°C ± 1°C and 60% ± 5%RH to exclude fluctuations in temperature and humidity. The total cultivation period was 22 days, divided into three key observation stages: Germination (Days 0–7), focusing on seed sprouting and seedling establishment; Vegetative (Days 8–15), monitoring rapid stem elongation; and Reproductive (Days 16–22), observing plant stabilization. The core of the experiment was comparing plant height between the drip irrigation treatment group and the flood irrigation control group, with other conditions (light, growing medium, etc.) kept consistent across groups. Plant height, the primary evaluation index, was measured multiple times at each stage by precisely recording the vertical distance from the medium surface to the highest natural point of the plant using a ruler, followed by calculating the average plant height for each treatment group. Through comparative analysis of plant height data and growth trends between the drip irrigation and control groups within and across stages, this demonstration visually demonstrates the specific impacts of drip irrigation technology on wheatgrass growth rate, developmental progression at each stage, and final plant height, providing direct growth‐response evidence for evaluating the application efficacy of drip irrigation.

## Author Contributions

C. H. and H. W. guided the project. H. W., C. H., X. Z., and D.C. conceived the idea and designed the experiment. D. C. and X. Z., fabricated the flexible electromagnetic actuator. D. C. and M. Z. performed the experiments and measurements. M. Z., K. L., Q. Z., Y. L., C. H., and H. W. revised the manuscript. All authors analyzed the experimental data, drew the figures and prepared the manuscript. All authors discussed the results and reviewed the manuscript.

## Conflicts of Interest

The authors declare no conflicts of interest.

## Supporting information




**Supporting File**: advs73958‐sup‐0001‐SuppMat.docx.

**Supporting File**: advs73958‐sup‐0002‐MovieS1.mp4.

**Supporting File**: advs73958‐sup‐0003‐MovieS2.mp4.

**Supporting File**: advs73958‐sup‐0004‐MovieS3.mp4.

## Data Availability

The data that support the findings of this study are available from the corresponding authors upon reasonable request.

## References

[1] P. Mehta , S. Siebert , M. Kummu , et al., “Half of Twenty‐First Century Global Irrigation Expansion Has Been in Water‐stressed Regions,” Nature Water 2 (2024): 254–261.

[2] J. Martínez‐Valderrama , J. Olcina , G. Delacámara , E. Guirado , and F. T. Maestre , “Complex Policy Mixes are Needed to Cope With Agricultural Water Demands under Climate Change,” Water Resources Management 37 (2023): 2805–2834.

[3] K. Schoengold and D. Zilberman , “The Economics of Water, Irrigation, and Development,” Handbook of Agricultural Economics 3 (2007): 2933–2977.

[4] Q. Ju , L. Du , C. Liu , and S. Jiang , “Water Resource Management for Irrigated Agriculture in China: Problems and Prospects,” Irrigation and Drainage 72 (2023): 854–863.

[5] P. Yang , L. Wu , M. Cheng , et al., “Review on Drip Irrigation: Impact on Crop Yield, Quality, and Water Productivity in China,” Water 15 (2023): 1733.

[6] M. N. Anjum M. J. M. Cheema , F. Hussain , and R.‐S. Wu , “Precision Irrigation: Challenges and Opportunities,” in Precision Agriculture: Evolution, Insights and Emerging Trends, ed. Q. U. Zaman and R. M. Malik (Elsevier, 2023), 85–101.

[7] B. Jayant , K. Dahiya , A. Rukhiyar , R. Raj , and R. K. Meena , “A Review of the Drip Irrigation System,” Journal of Engineering Research and Application 1 (2022): 22–29.

[8] M. K. Meriç , “Implementation of a Wireless Sensor Network for Irrigation Management in Drip Irrigation Systems,” Scientific Reports 15 (2025): 14157. 40269065 10.1038/s41598-025-97303-w PMC12019383

[9] H. Guo and S. Li , “A Review of Drip Irrigation's Effect on Water, Carbon Fluxes, and Crop Growth in Farmland,” Water 16 (2024): 2206.

[10] Y. Wang , Y. Zhang , W. Wang , et al., “A Review of Optimal Design for Large‐Scale Micro‐Irrigation Pipe Network Systems,” Agronomy 13 (2023): 2966.

[11] X. Wang , C. Zhang , and G. Li , “Improving Drip Irrigation Uniformity by Boosting the Hydraulic Performance of Drip Lateral Pressure Regulators,” International Journal of Agricultural and Biological Engineering 17 (2024): 185–192.

[12] Z. Liang , T. Zou , X. Liu , G. Liu , and Z. Liu , “Collaborative Operation and Application Influence of Sprinkler Drip Irrigation: A Systematic Progress Review,” International Journal of Agricultural and Biological Engineering 16 (2023): 12–27.

[13] Z. Ren , B. Lv , C. Shi , and Y. Wang , “Numerical Simulation and Optimization Analysis of a New Percolation Irrigator,” in *2022 3rd International Conference on Intelligent Design* *(ICID)* (IEEE, 2022), 213–217.

[14] Z. Zhu , G. Guan , X. Tian , S. M. Hashemy Shahdany , and K. Wang , “The Integrator Dual‐Delay Model for Advanced Controller Design of the Open Canal Irrigation Systems With Multiple Offtakes,” Computers and Electronics in Agriculture 205 (2023): 107616.

[15] M. Benzaouia , B. Hajji , A. Mellit , and A. Rabhi , “Fuzzy‐IoT Smart Irrigation System for Precision Scheduling and Monitoring,” Computers and Electronics in Agriculture 215 (2023): 108407.

[16] D. Xie , L. Chen , L. Liu , L. Chen , and H. Wang , “Actuators and Sensors for Application in Agricultural Robots: A Review,” Machines 10 (2022): 913.

[17] Y. Roh , D. Lim , M. Kang , J. Cho , S. Han , and S. H. Ko , “Rigidity‐Tunable Materials for Soft Engineering Systems,” Advanced Engineering Materials 26 (2024): 2400563.

[18] A. Sarker , T. U. Islam , and M. R. Islam , “A Review on Recent Trends of Bioinspired Soft Robotics: Actuators, Control Methods, Materials Selection, Sensors, Challenges, and Future Prospects,” Advanced Intelligent Systems 7 (2025): 2400414.

[19] M. Amini Najafabadi , R. Fatahi Nafchi , H. Salami , H. R. Vanani , and K. Ostad‐Ali‐Askari , “Effect of Different Managements With Drip Irrigation (Tape),” Applied Water Science 13 (2023): 37.

[20] J. Petit , S. M. García , B. Molle , R. Bendoula , and N. Ait‐Mouheb , “Methods for Drip Irrigation Clogging Detection, Analysis and Understanding: State of the Art and Perspectives,” Agricultural Water Management 272 (2022): 107873.

[21] A. Reda , M. A. Shahin , and P. Montague , “Review of Material Selection for Corrosion‐Resistant Alloy Pipelines,” Engineering Sciences 33 (2025): 1373.

[22] M. Tudi , L. Yang , J. Yu , et al., “Leaching Characteristics and Potential Risk of Heavy Metals from Drip Irrigation Pipes and Mulch Substrate in Agricultural Ecosystems,” Science of The Total Environment 882 (2023): 163573. 37076001 10.1016/j.scitotenv.2023.163573

[23] K. Zhao , H. Li , J. Ji , et al., “Pressure‐Stabilized Flexible End‐Effector for Selective Picking of Agaricus Bisporus,” Agriculture 13 (2023): 2256.

[24] Y. Yang , Y. Wu , C. Li , X. Yang , and W. Chen , “Flexible Actuators for Soft Robotics,” Advanced Intelligent Systems 2 (2020): 1900077.

[25] T. Jin and X. Han , “Robotic Arms in Precision Agriculture: A Comprehensive Review of the Technologies, Applications, Challenges, and Future Prospects,” Computers and Electronics in Agriculture 221 (2024): 108938.

[26] R. R. Shamshiri , E. Navas , V. Dworak , F. A. Auat Cheein , and C. Weltzien , “A Modular Sensing System With CANBUS Communication for Assisted Navigation of an Agricultural Mobile Robot,” Computers and Electronics in Agriculture 223 (2024): 109112.

[27] P. G. Steeneken , E. Kaiser , G. J. Verbiest , and M.‐C. ten Veldhuis , “Sensors in Agriculture: Towards an Internet of Plants,” Nature Reviews Methods Primers 3 (2023): 60.

[28] C. Prakash , L. P. Singh , A. Gupta , and S. K. Lohan , “Advancements in Smart Farming: A Comprehensive Review of IoT, Wireless Communication, Sensors, and Hardware for Agricultural Automation,” Sensors and Actuators A: Physical 362 (2023): 114605.

[29] L. Wang , J. Zhuo , J. Peng , H. Dong , S. Jiang , and Y. Shi , “A Stretchable Soft Pump Driven by a Heterogeneous Dielectric Elastomer Actuator,” Advanced Functional Materials 34 (2024): 2411160.

[30] J. Qin , W. Duan , S. Zou , Y. Chen , W. Huang , and L. Rosa , “Global Energy Use and Carbon Emissions from Irrigated Agriculture,” Nature Communications 15 (2024): 3084. 10.1038/s41467-024-47383-5 PMC11006866 38600059

[31] I. A. Lakhiar , H. Yan , C. Zhang , et al., “A Review of Precision Irrigation Water‐Saving Technology Under Changing Climate for Enhancing Water Use Efficiency, Crop Yield, and Environmental Footprints,” Agriculture 14 (2024): 1141.

[32] H. Zhao , R. Wen , L. Zhang , et al., “Magneto‐Controlled Tubular Liquid Actuators with Pore Engineering for Liquid Transport and Regulation,” Advanced Science 11 (2024): 2406325. 39137359 10.1002/advs.202406325 PMC11497001

[33] K. Shi , T. Lu , W. Zheng , X. Zhang , and L. Zhangzhong , “A Review of the Category, Mechanism, and Controlling Methods of Chemical Clogging in Drip Irrigation System,” Agriculture 12 (2022): 202.

[34] R. Benameur , A. Dahane , B. Kechar , and A. E. H. Benyamina , “An Innovative Smart and Sustainable Low‐Cost Irrigation System for Anomaly Detection Using Deep Learning,” Sensors 24 (2024): 1162. 38400320 10.3390/s24041162 PMC10892454

[35] W. Gu , F. Wang , S. Siebert , et al., “The Asymmetric Impacts of International Agricultural Trade on Water Use Scarcity, Inequality and Inequity,” Nature Water 2 (2024): 324–336.

[36] Y. Xing and X. Wang , “Precision Agriculture and Water Conservation Strategies for Sustainable Crop Production in Arid Regions,” Plants 13 (2024): 3184. 39599396 10.3390/plants13223184 PMC11598231

[37] S. Liu , C. Zhang , T. Shen , et al., “Efficient Agricultural Drip Irrigation Inspired by Fig Leaf Morphology,” Nature Communications 14 (2023): 5934. 10.1038/s41467-023-41673-0 PMC10518012 37741843

[38] L. Rosa , D. D. Chiarelli , M. C. Rulli , J. Dell'Angelo , and P. D'Odorico , “Global Agricultural Economic Water Scarcity,” Science Advances 6 (2020): aaz6031. 10.1126/sciadv.aaz6031 PMC7190309 32494678

[39] R. Hashem , M. Stommel , L. K. Cheng , and W. Xu , “Design and Characterization of a Bellows‐Driven Soft Pneumatic Actuator,” IEEE/ASME Transactions on Mechatronics 26 (2020): 2327–2338.

[40] Z. Qiu , S. Zhang , Y. Xue , et al., “An Empirical Model of Soft Bellows Actuator,” Scientific Reports 14 (2024): 28681. 39562794 10.1038/s41598-024-79084-w PMC11577089

[41] S. Hermann , P. Butaud , G. Chevallier , J.‐F. Manceau , and C. Espanet , “Magnetic and Dynamic Mechanical Properties of a Highly Coercive MRE Based on NdFeB Particles and a Stiff Matrix,” Smart Materials and Structures 29 (2020): 105009.

[42] S. Liang , X. Shao , Y. Que , et al., “Recent Advances in Mechanical Properties of Sintered NdFeB Magnets,” Journal of Alloys and Compounds 1003 (2024): 175689.

[43] N. Salidkul , P. Thongbai , and S. Pinitsoontorn , “Enhanced Magnetic Properties and Densification of SrFe_12_O_19_/Fe_3_O_4_ Hard/Soft Composites via Cold Sintering Process,” Journal of Science: Advanced Materials and Devices 10 (2025): 100900.

[44] P. Cui , W. Zhu , H. Ji , H. Chen , C. Hang , and M. Li , “Analysis and Optimization of Induction Heating Processes by Focusing the Inner Magnetism of the Coil,” Applied Energy 321 (2022): 119316.

[45] Q.‐Y. Zeng , G.‐X. Su , A.‐S. Song , et al., “High‐Quality‐Factor Viscoelastic Nanomechanical Resonators From Moiré Superlattices,” Nature Communications 16 (2025): 3793. 10.1038/s41467-025-58981-2 PMC12015328 40263349

[46] Z. Liao , M. Hossain , X. Yao , R. Navaratne , and G. Chagnon , “A Comprehensive Thermo‐Viscoelastic Experimental Investigation of Ecoflex Polymer,” Polymer Testing 86 (2020): 106478.

[47] Y. Guo , D. Wang , R. Xing , et al., “Magnetic Properties and Magnetoimpedance in FINEMET/NdFeB Composite Ribbons,” Materials Research Bulletin 190 (2025): 113507.

[48] M. A. Darwesh , O. H. El‐Shiaty , M. M. Farahat , and A. S. Ibrahim , “Response of Monstera Deliciosa Liebm. To Plant Spacing and Nitrogen Sources Fertilizer,” Egyptian Journal of Agricultural Sciences 62 (2011): 81–92.

[49] F. Yang , H. Li , and Y. Jiang , “A Review of Pressure Regulation Technologies for Irrigation Pipeline Systems,” Agriculture 15, no. 14 (2025): 1528.

[50] H. Steppuhn , P. G. Jefferson , A. D. Iwaasa , and J. G. McLeod , “AC Saltlander Green Wheatgrass,” Canadian Journal of Plant Science 86 (2006): 1161–1164.

[51] H. Yin , Y. Cao , B. Marelli , X. Zeng , A. J. Mason , and C. Cao , “Soil Sensors and Plant Wearables for Smart and Precision Agriculture,” Advanced Materials 33 (2021): 2007764. 10.1002/adma.202007764 33829545

[52] Ministry of Water Resources of the People's Republic of China , “Annual Precipitation in Each Province and Municipality,” 2023, http://www.mwr.gov.cn/sj/tjgb/szygb/202406/t20240614_1713318.html.

[53] Water Resources Department of Xinjiang Uygur Autonomous Region , “Annual Precipitation in Each Region,” 2023, https://slt.xinjiang.gov.cn/xjslt/c114491/zfxxgk_list.shtml.

[54] H. Zhou , P. Zhou , H. Ping , et al., “Analysis of Agricultural Irrigation Water‐Using Coefficient in Xinjiang Arid Region,” Transactions of the Chinese Society of Agricultural Engineering 29 (2013): 100–107.

[55] X. Hou , J. Fan , F. Zhang , et al., “Determining Water Use and Crop Coefficients of Drip‐Irrigated Cotton in South Xinjiang of China Under Various Irrigation Amounts,” Industrial Crops and Products 176 (2022): 114376.

[56] X.‐C. Fan , W.‐Q. Wang , R.‐J. Shi , and Z.‐J. Cheng , “Hybrid Pluripotent Coupling System with Wind and Photovoltaic‐Hydrogen Energy Storage and the Coal Chemical Industry in Hami, Xinjiang,” Renewable and Sustainable Energy Reviews 72 (2017): 950–960.

[57] Y. Song , D. Xue , B. Ma , S. Xia , and H. Ye , “Farming in Arid Areas Depletes China's Water,” Science 379 (2023): 651. 10.1126/science.adg4780 36795840

[58] S. E. Null , H. Zeff , J. Mount , et al., “Storing and Managing Water for the Environment is More Efficient Than Mimicking Natural Flows,” Nature Communications 15 (2024): 5462. 10.1038/s41467-024-49770-4 PMC11211385 38937466

