# Supplementary material for: Water‐Efficient Smart Drip Irrigation Enabled by a Low‐Power and High‐Efficiency Flexible Electromagnetic Actuator
Source: Adv Sci (Weinh). 2026 Jan 21;13(18):e14950. doi: 10.1002/advs.202514950 (PMC13042495; doi:10.1002/advs.202514950)
Supplement: Supplementary file 1 — Supporting File: advs73958‐sup‐0001‐SuppMat.docx. [file ADVS-13-e14950-s003.docx]

**Supporting Information**

**Water-efficient smart drip irrigation enabled by a low-power and high-efficiency flexible electromagnetic actuator**

Duo Chen^1, 4^, Xubin Zhu^1, 4^, Minwei Zhang^2^, Kerui Li^1^, Qinghong Zhang^3^, Yaogang Li^3^, Chengyi Hou^1, 2*^, Hongzhi Wang^1*^

*Corresponding author: Hongzhi Wang ([wanghz@dhu.edu.cn](mailto:wanghz@dhu.edu.cn)), Chengyi Hou ([hcy@dhu.edu.cn](mailto:hcy@dhu.edu.cn))

**This file includes:**

**Supplementary Figures**

**Supplementary Fig. 1** Actuation mechanism of electromagnetically driven flexible actuator and corrugated matrix deformation.

**Supplementary Fig. 2** Preparation processes and physical morphology comparison of three flexible magnetic corrugated matrices.

**Supplementary Fig. 3** SEM and elemental distribution characterization of Fe₃O₄-silicone composites.

**Supplementary Fig. 4** Magnetic response of magnetized NdFeB-silicone corrugated matrix.

**Supplementary Fig. 5** Magnetic adsorption performance and dynamic response of Fe₃O₄-silicone flexible corrugated matrix.

**Supplementary Fig. 6** Different irrigation methods for plants.

**Supplementary Fig. 7** Time-lapse observation of wheatgrass growth dynamics during germination stage under different irrigation methods.

**Supplementary Fig. 8** Dynamic effects and cultivation outcomes of electromagnetically driven flexible actuators irrigation modes on wheatgrass growth.

**Supplementary Fig. 9** Environmental regulation setup and experimental physical images during wheatgrass growth cycle.

**Supplementary Fig. 10** Agricultural water consumption and groundwater resources distribution in China.

**Supplementary Fig. 11** Water consumption by sector and annual water resources heatmap of second-level administrative divisions in Xinjiang, China.

**Supplementary Tables**

**Supplementary Table 1** Analysis of Common Crop Growth Stage Water Demand Patterns and Adaptability of the Flexible Electromagnetic Actuator

**Supplementary Table 2** Analysis of Common Crop Growth Stage Water Demand Patterns and Flexible Electromagnetic Actuator Adaptation Schemes

**Supplementary Tables 3** Laboratory drive water saving rates and water saving data for the Xinjiang region during the growth cycle

**Supplementary Table 4** Laboratory drive energy consumption and total energy consumption data for the Xinjiang region during the growth cycle

**Supplementary Notes**

**Supplementary Note 1** Dynamic Rheological Model of Ecoflex Silicone

**Supplementary Note 2** Flow control calculations for flexible electromagnetic actuator

**Supplementary Note 3** Cultivation of plant growth explanatory notes

**Supplementary Note 4** Environmental correction coefficients for water conservation and energy consumption in the Xinjiang region.

**Supplementary Figures**


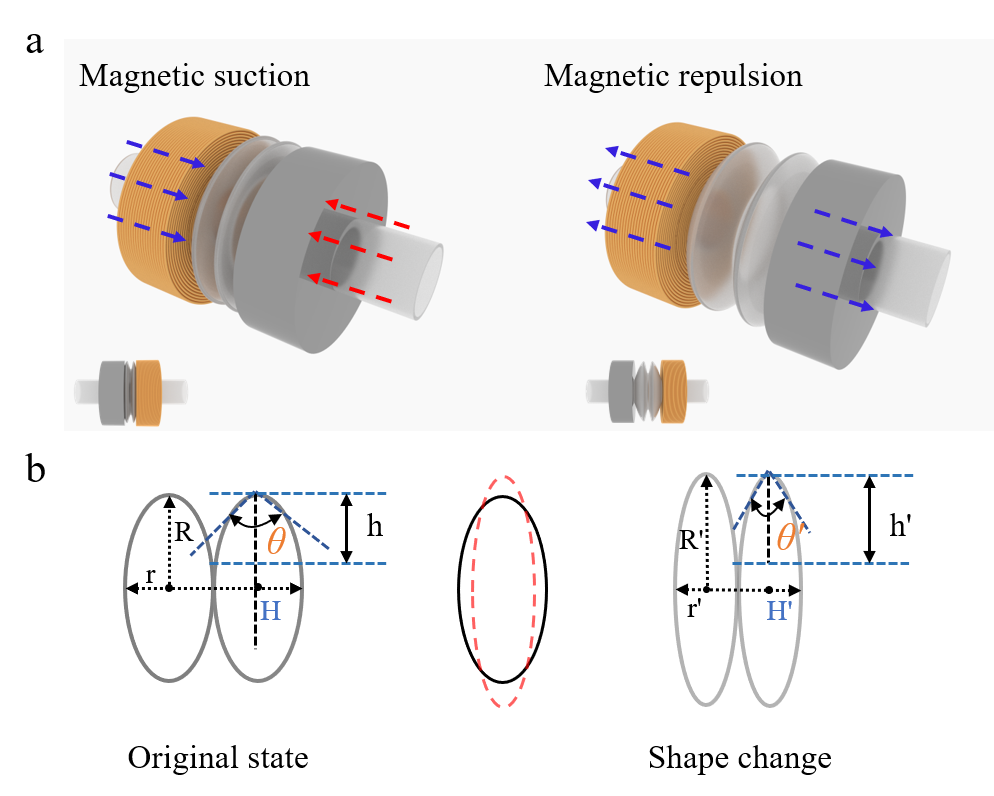


**Supplementary Fig. 1 Actuation mechanism of electromagnetically driven flexible actuator and corrugated matrix deformation. a** When current is applied to the flexible electromagnetic actuator forward excitation induces mutual attraction between the magnet and induction coil, generating compressive force; reverse excitation triggers mutual repulsion, enabling the flexible matrix to revert to its original shape. This periodic deformation achieves continuous water pumping. **b** The flexible matrix is composed of two approximate ellipsoids. When subjected to compression, the matrix deforms, and the volume change caused by this deformation can be quantitatively calculated using parameters^1,2^.


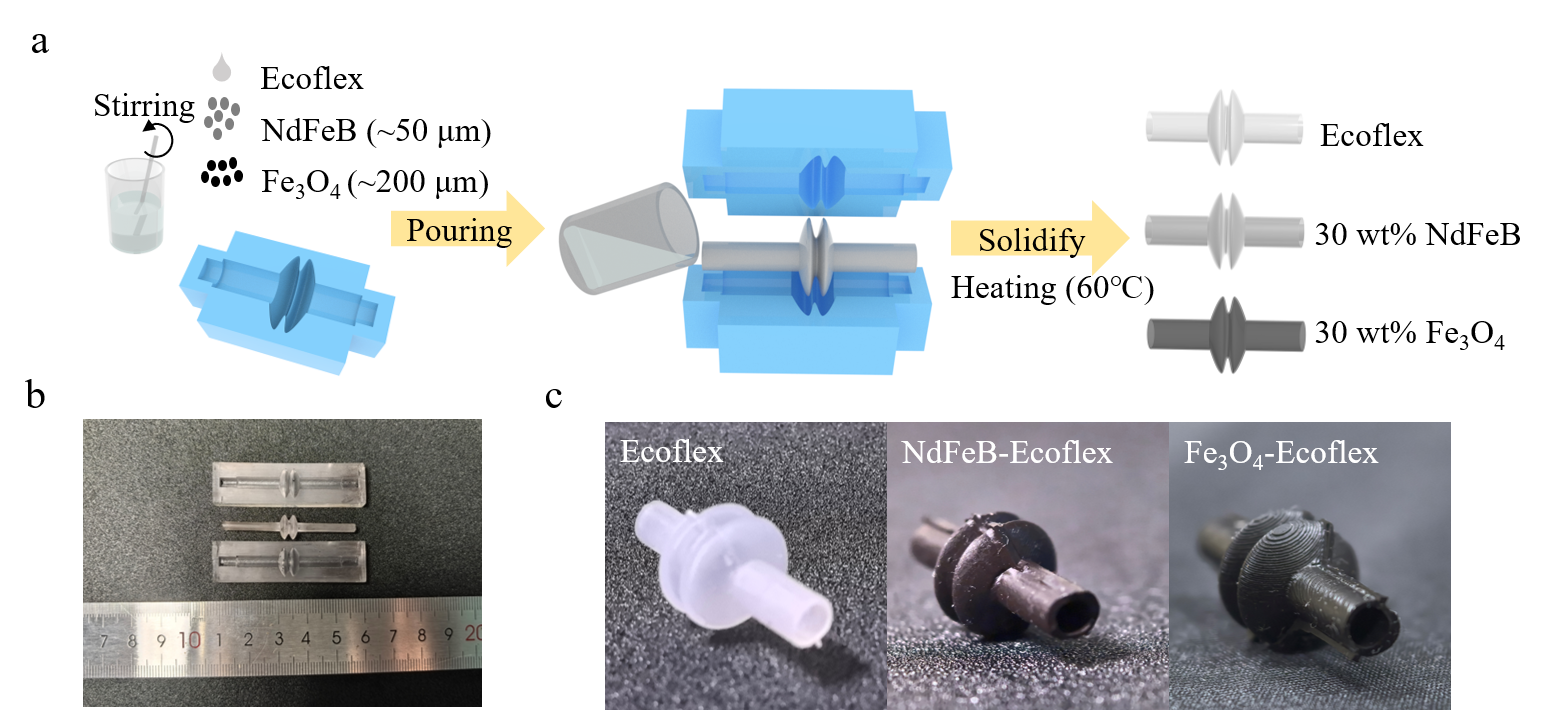


**Supplementary Fig. 2 Preparation processes and physical morphology comparison of three flexible magnetic corrugated matrices**. **a** Schematic diagram of the grouting molding process for flexible silicone matrices. Liquid silicone is vacuum-degassed before being injected into a custom corrugated mold, forming a smooth-surfaced flexible matrix after curing. For Fe_3_O_4_-silicone composites, magnetic particles are mechanically mixed with silicone prepolymer before mold injection and curing; the process for NdFeB-silicone composites is identical. **b** Physical photographs of the fabricated models. **c** The silicone flexible matrix without magnetic particles exhibits good luster and a milky translucent appearance. Before magnetization, flexible matrices containing magnetic particles show metallic sheen on their surfaces.


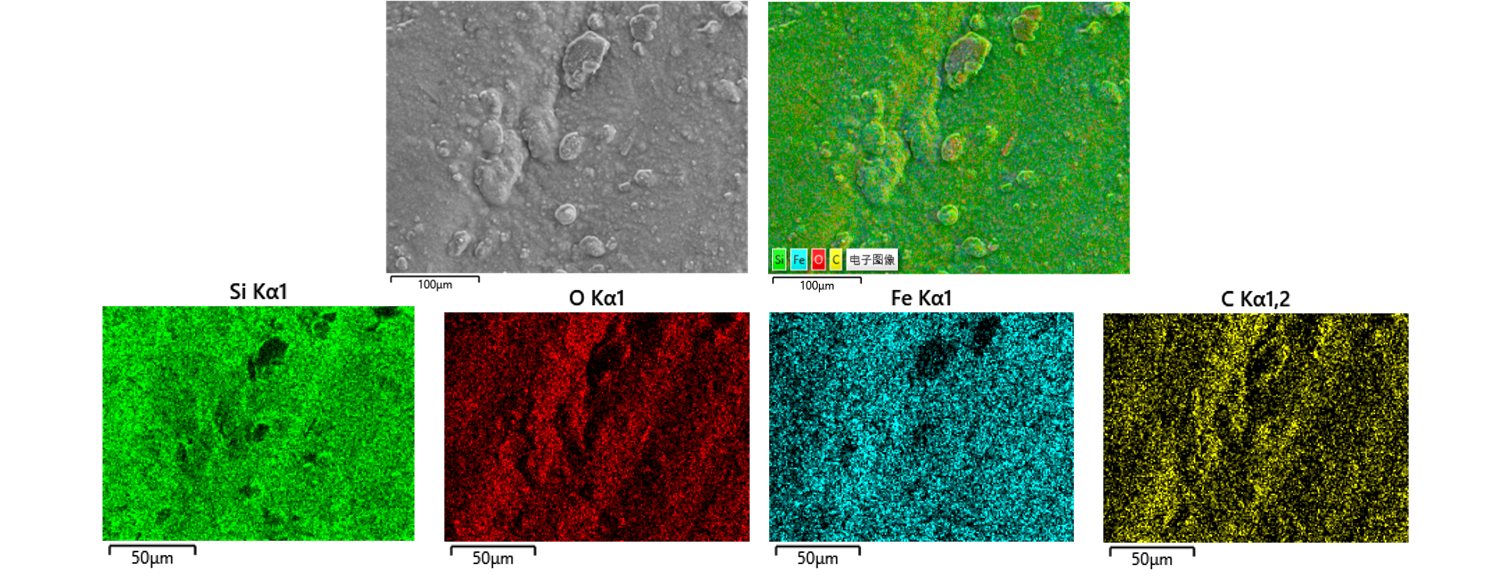


**Supplementary Fig. 3** **SEM and elemental distribution characterization of Fe_3_O_4_-silicone composites.** Cross-sectional SEM images reveal Fe_3_O_4_ particles embedded within the silicone matrix, with partial agglomeration and occasional defects. Elemental mapping confirms interpenetration between the silicone matrix and Fe_3_O_4_ particles.


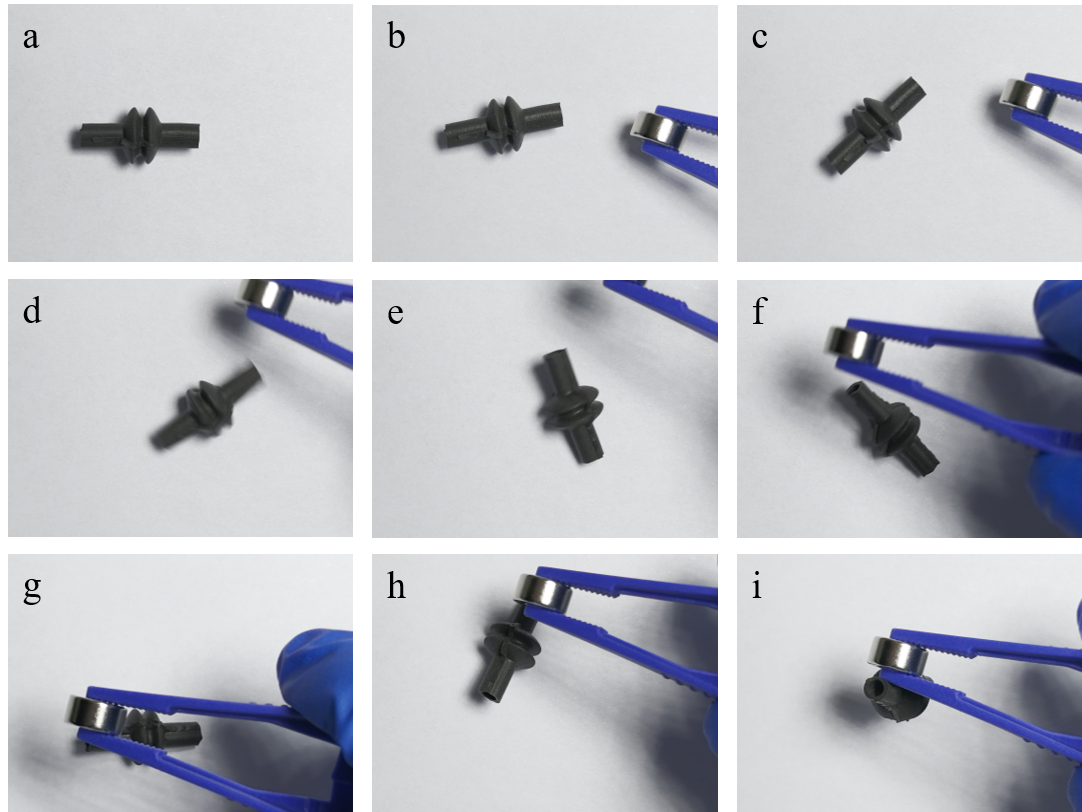


**Supplementary Fig. 4** **Magnetic response of magnetized NdFeB-silicone corrugated matrix. a-i** When a magnet approaches the magnetized NdFeB-silicone flexible corrugated matrix, the two ends of the matrix form N and S poles. When the south (S) pole of an external magnet approaches the matrix’s S pole, like-pole repulsion induces radial rotation and magnetic repulsion. Conversely, when the north (N) pole of an external magnet approaches the matrix’s S pole, opposite-pole attraction directly pulls the matrix toward the magnet, demonstrating excellent magnetic responsiveness after magnetization.


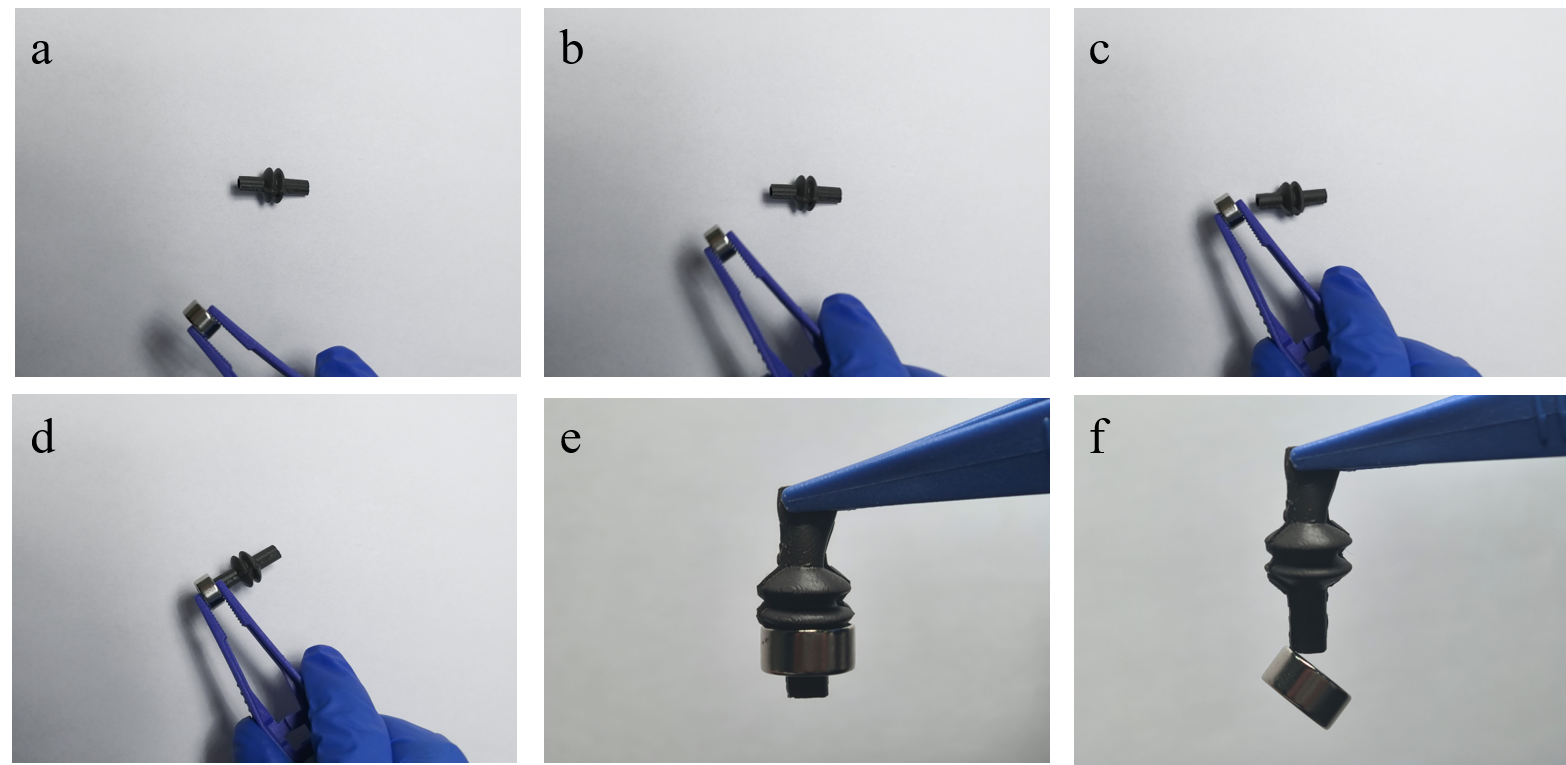


**Supplementary Fig. 5 Magnetic adsorption performance and dynamic response of Fe_3_O_4_-silicone flexible corrugated matrix**. **a-f**: Attraction behavior of the Fe_3_O_4_ flexible matrix to a magnet. When an external magnet (N pole) approaches the Fe_3_O_4_-silicone matrix, the matrix is rapidly magnetized due to its soft magnetic properties, forming instantaneous magnetic poles on the surface and generating strong attractive force. The matrix can be attracted and suspended through a hollow magnet, or directly adsorb and suspend a 10 g magnet, demonstrating its robust magnetic adsorption capacity.


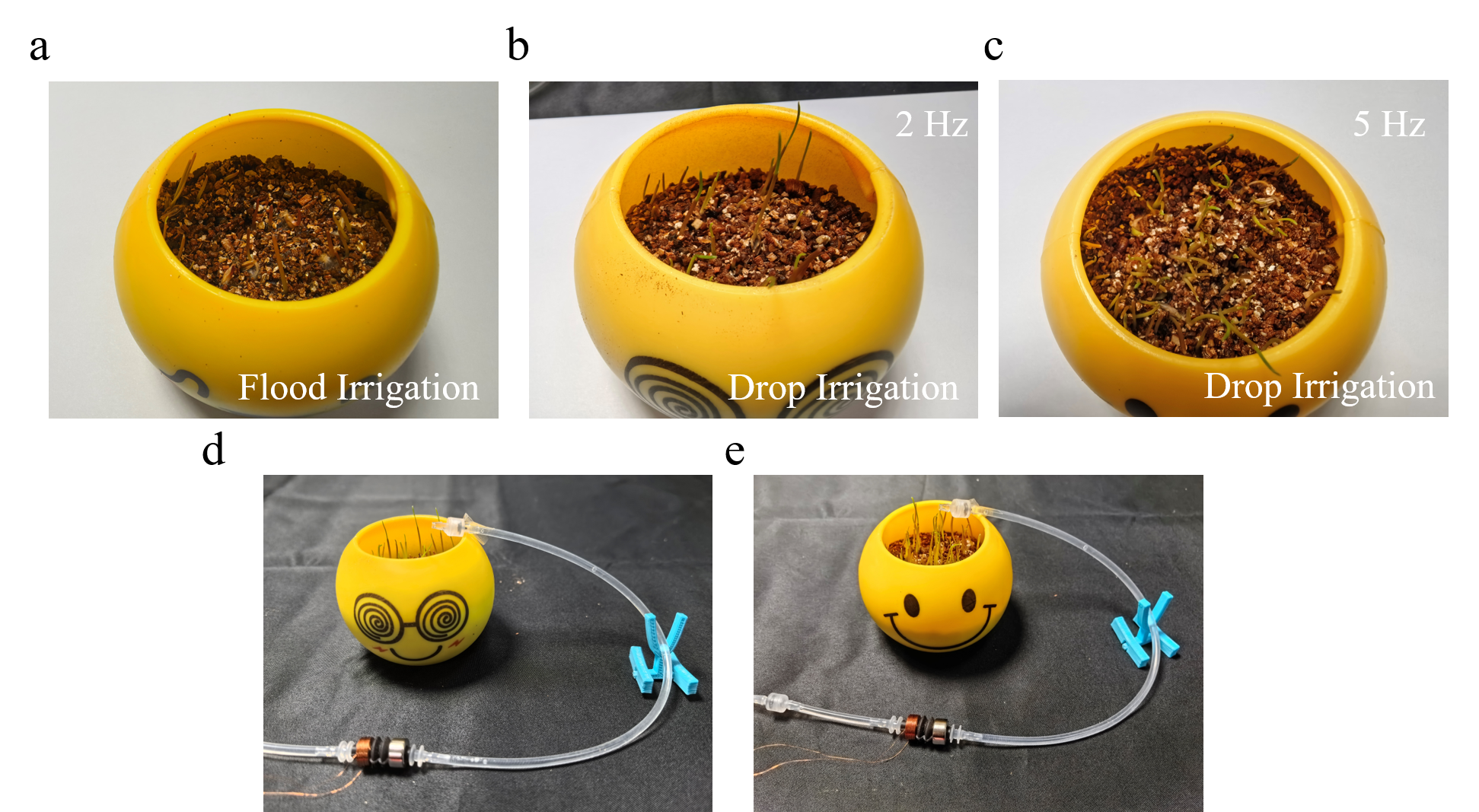


**Supplementary Fig. 6 Different irrigation methods for plants**. **a-c**: Two days after sowing, wheatgrass seeds successfully germinated under both flood irrigation and dropwise irrigation conditions, indicating that both methods provide sufficient water to support basic germination in the early stage. **d-e**: Growth status comparison of seedlings under 2Hz low-frequency dropwise irrigation and 5Hz high-frequency dropwise irrigation, visually comparing the effects of different irrigation frequencies on wheatgrass germination progression and early seedling growth.


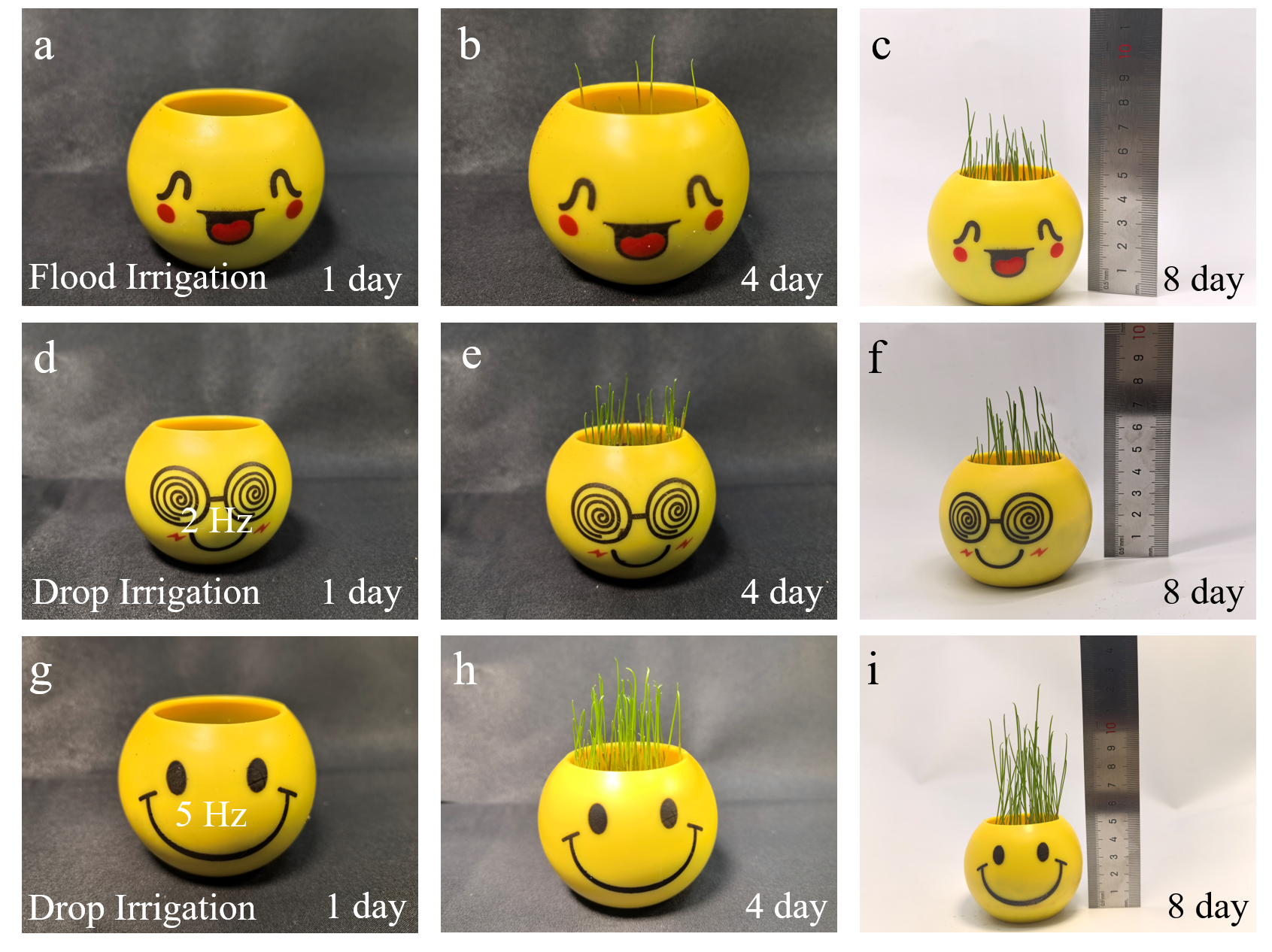


**Supplementary Fig. 7** **Time-lapse observation of wheatgrass growth dynamics during germination stage under different irrigation methods. a-i**: Sequential images from initial seed germination to plant height formation under flood irrigation, 2Hz dropwise irrigation, and 5Hz dropwise irrigation, showing differences in early seedling growth among the three treatments. Observations include plant height, leaf expansion, and overall robustness changes under each irrigation regime.


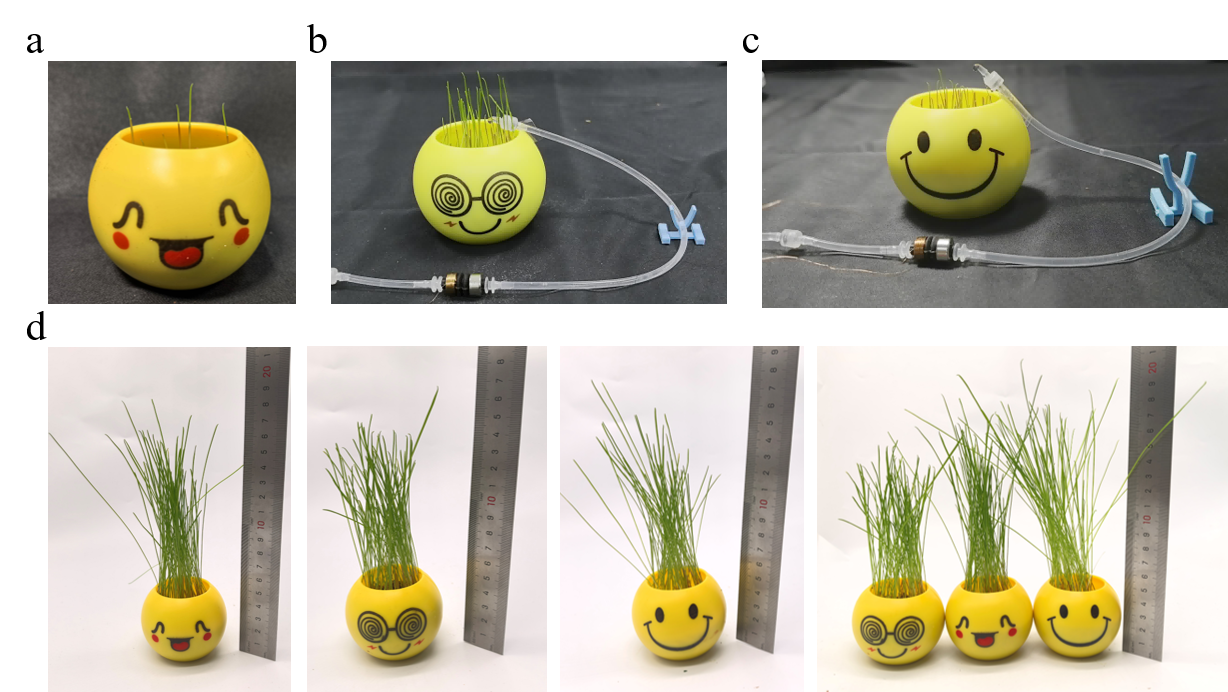


**Supplementary Fig. 8 Dynamic effects and cultivation outcomes of electromagnetically driven flexible actuators irrigation modes on wheatgrass growth**. **a** Flood irrigation: single injection of 8 mL water. **b** 2 Hz dropwise irrigation (1.6 mL/min): generating pulsed water droplets at 0.5 s intervals. **c** 5 Hz dropwise irrigation (3.6 mL/min): high-frequency pulses (0.2 s intervals) forming a continuous infiltration effect during cultivation. **d** Growth phenotype comparison of wheatgrass under three irrigation modes. After 18 days of cultivation, the 5 Hz group exhibited optimal growth, with significantly greater plant height than both the flood irrigation and 2 Hz groups.


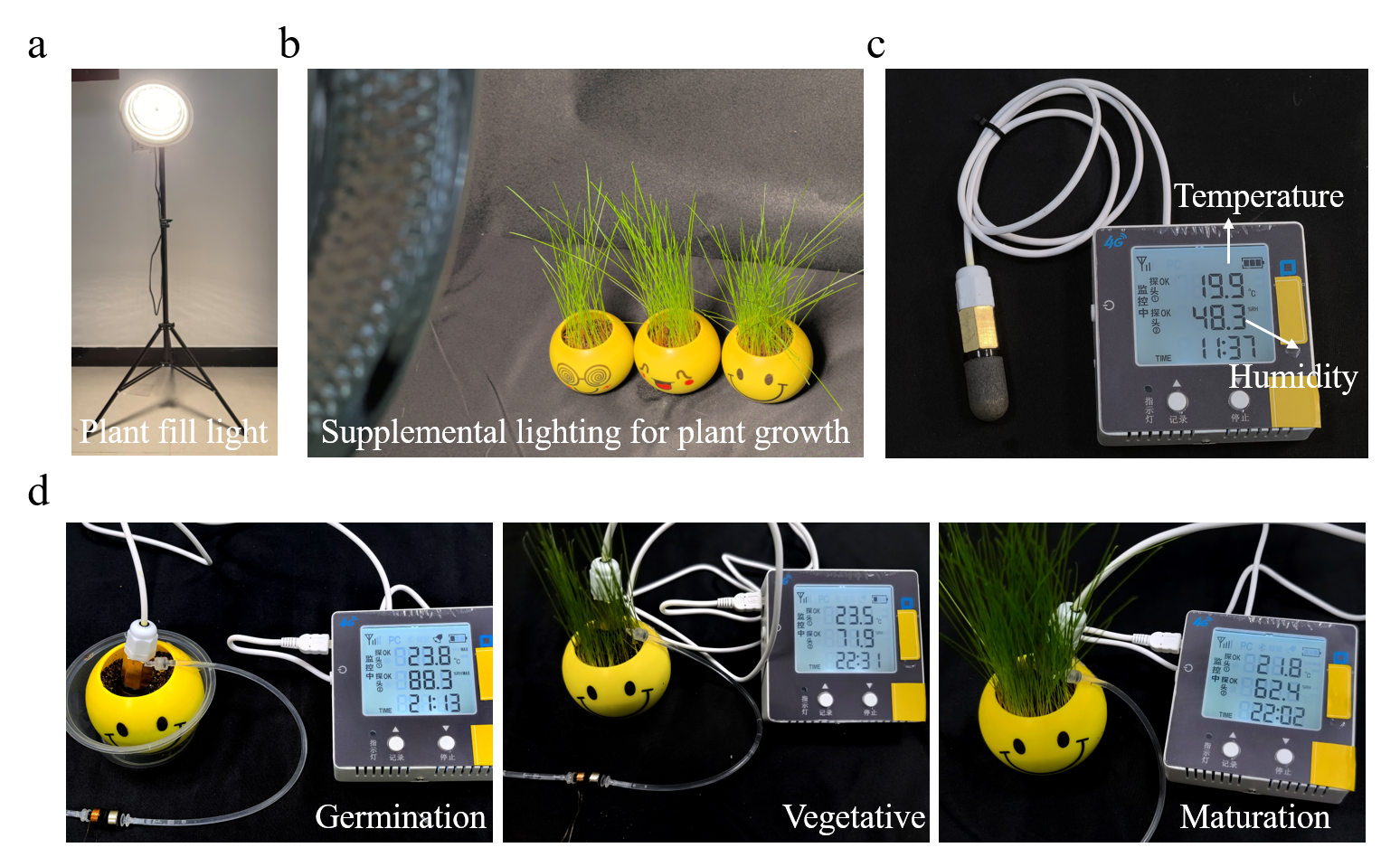


**Supplementary Fig. 9** **Environmental regulation setup and experimental physical images during wheatgrass growth cycle**. **a-b** Supplementary lighting during the vegetative and maturity stages: additional light was provided by plant growth lamps to enhance photosynthesis. **c-d** Thermohygrometer-controlled environmental regulation: throughout the growth cycle, watering was adjusted according to growth characteristics and soil moisture, with humidity maintained at 75-90 %RH during germination, 60-75 %RH during vegetative growth, and >50 %RH during maturity, ensuring optimal temperature and humidity conditions for each stage.


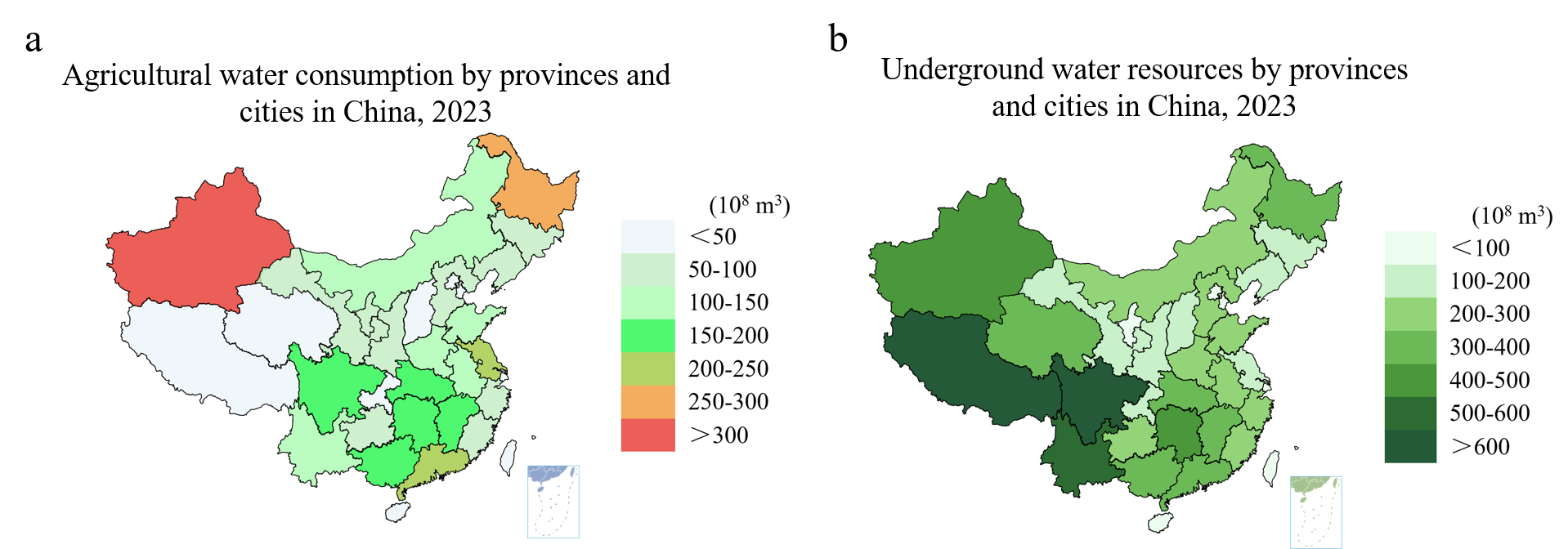


**Supplementary Fig. 10** **Agricultural water consumption and groundwater resources distribution in China. a** Provincial-level agricultural water consumption and **b** groundwater resources distribution in China. In 2023, national agricultural water consumption reached 367.24 billion m^3^, accounting for 62.2% of the total water usage. Arid regions in northern China heavily rely on groundwater for irrigation due to scarce precipitation, while humid southern regions primarily use surface water. Total groundwater resources amount to 780.71 billion m^3^, with Tibet (99.35 billion m^3^) and Sichuan (62.59 billion m^3^) boasting abundant reserves from alpine snowmelt and precipitation, though low development rates due to ecological constraints. Severe groundwater overexploitation occurs in the North China Plain and Northeast Black Soil Region, with Hebei and Henan experiencing continuous water table decline from agricultural use. As a typical arid region, Xinjiang consumes 52.79 billion m^3^ of agricultural water (14.4% of the national total), the highest among provinces. Its surface water relies on snowmelt from the Tianshan and Kunlun Mountains, while groundwater resources stand at 46.77 billion m^3^, with only 4.22 billion m^3^ of non-repeating utilisable volume. Over 80% of agricultural irrigation depends on groundwater, exacerbating ecological degradation and desertification in the Tarim River Basin. Despite an improved farmland irrigation water use efficiency coefficient (0.581), water conflicts intensify due to oasis expansion and cotton cultivation. Sustainable water management—via drip irrigation promotion, cropping structure adjustment, and inter-basin water transfer—is urgent to balance food security and ecological protection^3^.


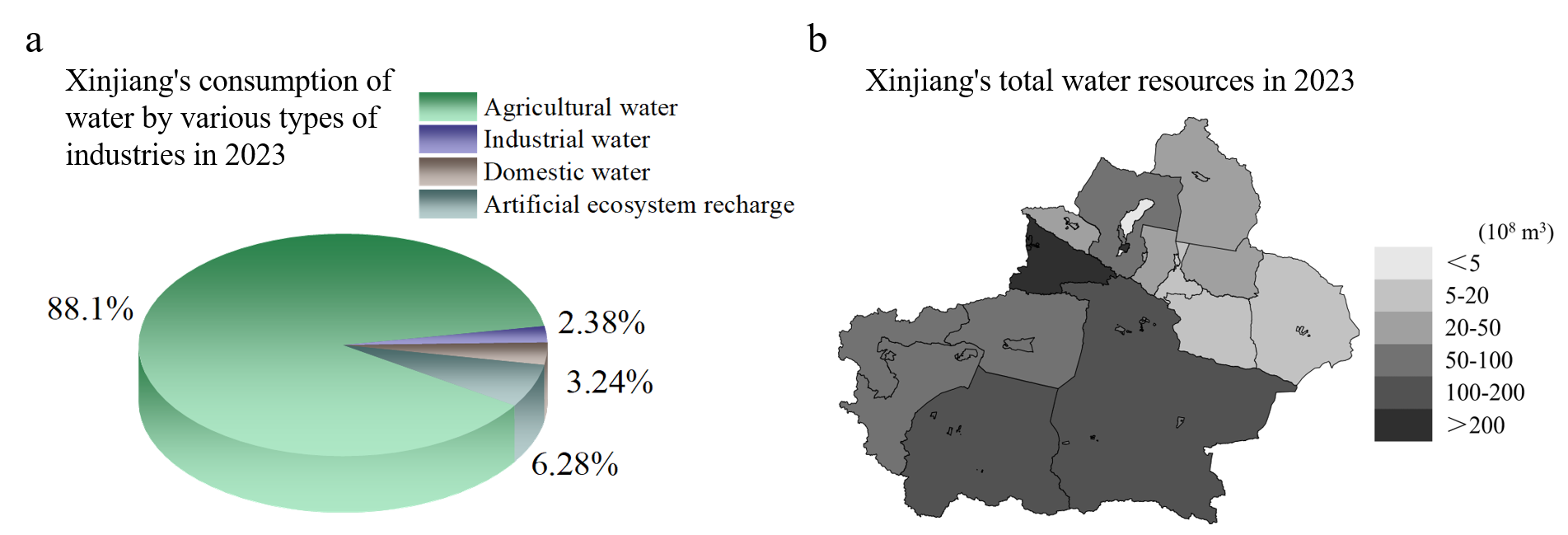


**Supplementary Fig. 11 Water consumption by sector and annual water resources heatmap of second-level administrative divisions in Xinjiang, China**. **a** Pie chart of sectoral water consumption in Xinjiang, China, in 2023 shows agricultural water use accounts for 88.1% (64.78 billion m^3^), with industry, domestic use, and ecological water replenishment comprising only 2.38%, 3.24%, and 6.28%, respectively, highlighting the region’s extreme dependence on water for agriculture. **b** Heatmap of annual water resources distribution across second-level administrative divisions reveals highly uneven availability: Ili Kazak Autonomous Prefecture (approximately 12 billion m^3^) in northern Xinjiang and Aksu Prefecture (approximately 9 billion m^3^) in southern Xinjiang form high-value zones due to Tianshan snowmelt recharge, while Turpan City (0.8 billion m^3^), Hami City (1 billion m^3^), and counties within the Taklamakan Desert face severe water scarcity. Of Xinjiang’s total water resources (86.83 billion m^3^), 95.2% is surface water (82.62 billion m^3^), but actual utilisable volume is less than 60% due to high evaporation and spatiotemporal imbalance. Severe groundwater overexploitation (extraction rate >80%) has caused ecological crises such as downstream Tarim River cutoff and Lop Nur drying^4^. Despite an improved farmland irrigation water use efficiency coefficient (0.581, slightly higher than the national average of 0.576), water conflicts intensify with oasis expansion and cotton cultivation. Sustainable water management—via inter-basin water transfer, cropping structure adjustment, and strict extraction controls—is urgent. Xinjiang’s water-saving practices and ecological restoration hold significant demonstration value for global arid-region water resource management.

**Supplementary Table 1** Analysis of Common Crop Growth Stage Water Demand Patterns and Adaptability of the Flexible Electromagnetic Actuator

| Comparison Dimension | Flexible Electromagnetic Actuator | Typical Commercial Irrigation Systems |
| --- | --- | --- |
| Working Principle | Magnetic-force coupling drives volumetric squeezing of flexible bellows | Solenoid Valve (on/off control)  Pressure-Compensating Emitter (mechanical pressure stabilization) |
| Drive Energy Consumption | Very Low (milliwatt level ~1 W)  Operating Condition: 50–200 mA @ <5V | Relatively High (several watts)  Solenoid Valve: ~2-10 W (12/24V DC or 220V AC); Small Pump: tens to hundreds of watts |
| Flow Control Range & Precision | Wide-Range, Continuously Adjustable (0.05~5.8 mL/min), controlled by adjusting current/frequency | Fixed or Limited Steps |
| System Pressure Requirement | Very Low (only to overcome pipeline resistance) | Relatively High (requires pressure for activation and stable pressure to ensure flow) |
| Core Advantages | Ultra-low power consumption, continuous and precise programmable flow, no high pressure required | Mature technology, high reliability, suitable for large-scale field applications, high degree of standardization |

Compared with commercially available counterparts^5-7^, the actuator proposed in this work exhibits intrinsic potential advantages in micropower-driven operation and precision control of milliliter-scale flow rates from a fundamental principle perspective. These advantages align well with the core requirements for miniaturized, distributed, and precisely controllable actuation units in precision and smart agriculture.

**Supplementary Table 2** Analysis of Common Crop Growth Stage Water Demand Patterns and Flexible Electromagnetic Actuator Adaptation Schemes

| Crop Type | Growth Stage | Stage Objective & Water Demand Characteristics | Flexible Electromagnetic Actuator Adaptation Scheme |
| --- | --- | --- | --- |
| Lettuce | Germination | Promote root establishment, keep surface root zone consistently moist but with small water volume to prevent damping-off | Low-frequency, micro-volume: 0.5-1 Hz, ~0.1 mL per pulse, maintaining slight moisture in root zone. |
|  | Vegetative | Rapid leaf expansion, peak water demand, requires stable and adequate water supply. | High-frequency, adequate volume: 3-5 Hz, ~0.3 mL per pulse. Stable supply to root zone, keeping leaf surface dry. |
|  | Maturation | Growth slows, water demand decreases, moderate water stress may improve quality. | Low-frequency, reduced volume: Return to 1-2 Hz, ~0.1 mL per pulse. Implement controlled water stress. |
| Tomato | Germination | Cultivate strong seedlings, moderate water demand, water fluctuations can lead to flowering disorders. | Stable medium-frequency: 1-2 Hz, ~0.2 mL per pulse. Provide uniform, controlled moisture. |
|  | Vegetative | Concurrent vegetative and reproductive growth, critical water period. Water deficit causes flower/fruit drop; excess causes leggy growth. | Precise regulation: Dynamically adjust between 2-4 Hz and 0.2-0.4 mL based on soil moisture feedback. |
|  | Maturation | Peak water demand, especially during fruit expansion. Late-stage controlled watering to increase sugar content. | Increase then decrease: Use 3-5 Hz high frequency during expansion, reduce to 1-2 Hz during ripening for deficit irrigation. |
| Cotton | Germination | Low water demand, moderate drought promotes root penetration. | Low-frequency induction: Use very low frequency (0.5-1 Hz) to create moderate drought signal, promoting deep roots. |
|  | Vegetative | Critical water period, extremely sensitive to water stress, must ensure adequate supply. | High-frequency, adequate volume: Use 4-5 Hz to ensure sufficient moisture in root zone, countering transpiration. |
|  | Maturation | Reduced water demand, moderate drought promotes boll opening and improves fiber quality. | Gradual reduction: Frequency gradually decreases from 2 Hz to 0.5 Hz, guiding controlled physiological maturation. |

This work presents proof-of-principle demonstration and preliminary positive evidence for flexible electromagnetic actuator-based precision drip irrigation. Its inherent programmability and broad-range controllability underpin its potential for universal deployment. While direct extrapolation of results from wheatgrass to other crops necessitates further experimental validation, the integrated analysis of existing data and mechanistic pathways illustrates the technology’s compatibility with common agricultural crop systems, indicating high feasibility and promising application outlook for synergistic water conservation and growth promotion^8-10^.

**Supplementary Table 3** Laboratory drive water saving rates and water saving data for the Xinjiang region during the growth cycle

| **growth phase** | **Laboratory Water Savings (m^3^/ha)** | **Laboratory Water Savings (%)** | **Xinjiang drip irrigation water volume (m^3^/ha)** | **Xinjiang diffuse irrigation water quantity (m^3^/ha)** | **Water saving in Xinjiang (m^3^/ha)** | **Water saving rate in Xinjiang (%)** |
| --- | --- | --- | --- | --- | --- | --- |
| **Germination** | 61.4 | 48.8 | 64.4 | 126 | 61.6 | 48.8 |
| **Vegetative** | 33.6 | 23.3 | 110.4 | 144 | 33.6 | 23.3 |
| **Maturation** | 77.7 | 61.7 | 48.3 | 126 | 77.7 | 61.7 |
| **In all** | **172.9** | **43.7** | **223.1** | **396** | **172.9** | **43.7** |

Note:

The actual water volume in Xinjiang is corrected based on the loss rate (15% loss for drip irrigation and 50% loss for diffuse irrigation)^11^.

$$Water saving rate=\frac{diffuse irrigation volume - drip irrigation volume}{diffuse irrigation volume}\times100\%$$

**Supplementary Table 4** Laboratory drive energy consumption and total energy consumption data for the Xinjiang region during the growth cycle

| **growth phase** | **Laboratory single plant energy consumption (Wh/plant)** | **Corrected energy consumption of single plant in Xinjiang (Wh/plant)** | **Laboratory total energy consumption (kWh/ha)** | **Total energy consumption in Xinjiang (kWh/ha)** | **Energy efficiency (m^3^/kWh)** |
| --- | --- | --- | --- | --- | --- |
| **Germination** | 0.466 | 0.496 | 124 | 496 | 0.124 |
| **Vegetative** | 0.799 | 0.851 | 39.5 | 851 | 0.0395 |
| **Maturation** | 0.351 | 0.373 | 208 | 373 | 0.208 |
| **In all** | 1.616 | 1.722 | 371.5 | 1,722 | 0.1004 |

Note:

Xinjiang corrected energy consumption Equation^12^:

$$Total Energy Consumption= Laboratory Energy Consumption\times1.25 (Environmental Compensation)\times0.85 (Intelligent Regulation)$$

$$Energy efficiency=\frac{water saving}{total energy consumption}$$

**Supplementary Notes**

**Supplementary Note 1** Dynamic Rheological Model of Ecoflex Silicone

$$G^{*}(w)=G^{'}(w)+iG^{''}(w)$$

$G^{*}(w)$: Complex shear modulus, characterizing the total stiffness of the material under alternating stress/strain.

$G^{'}(w):$ Storage modulus, characterizing the elastic part of the material, related to energy stored during deformation.

$G^{''}(w)$: Loss modulus, characterizing the viscous part of the material, related to energy dissipated as heat during deformation.

Dynamic rheological models illustrate that the viscoelasticity of silicone is delineated by its complex modulus. In the current work, the pure silicone actuator reaches a peak flow rate at 2 Hz, corresponding to the condition where the angular frequency ω and the material’s intrinsic characteristic relaxation time τ fulfill ω·τ = 1. Herein, the storage modulus ($G^{'}(w)$) and loss modulus ($G^{''}(w)$') attain an optimal balance: elastic deformation is sufficiently developed, while viscous dissipation remains relatively minimal. This synergistic effect ensures effective periodic pumping strokes and stable flow output^13^.

**Supplementary Note 2 Flow control calculations for flexible electromagnetic actuator** The flexible electromagnetic actuator generates fluid output through periodic deformation of the flexible corrugated matrix, induced by magnetic interaction between the excitation field (via current I) and the embedded magnet. Flow rate control involves multiparameter coupling of electromagnetic actuation, geometric deformation, and fluid mechanics (Supplementary Fig. 1).

The flow rate Q fundamentally depends on the product of single-deformation volume ΔV and vibration frequency f, where ΔV is determined by both the bellows' geometric parameters and the deformation magnitude^1,2^:

The effective fluid channel area of the annular cross-section is (where R denotes the outer radius and r denotes the inner radius):

$$\begin{aligned} \pi\left（ R^{2}-r^{2} \right）\#\left（ 1 \right） \end{aligned}$$

The effective vertical component of deformation depth d is (where θ denotes the deformation angle):

$$\begin{aligned} d\sin\theta\#\left（ 2 \right） \end{aligned}$$

Incorporating the deformation proportionality coefficient α (where 0 ≤ α ≤ 1, representing the ratio of actual deformation under electrical actuation, with m as the linear coefficient):

$$\begin{aligned} \alpha=m\cdot I\#\left（ 3 \right） \end{aligned}$$

The deformation volume is:

$$\begin{aligned} \Delta V=\pi\left（ R^{2}-r^{2} \right）\cdot\left（ \alpha d\sin\theta\right）\cdot\frac{H}{R}\#\left（ 4 \right） \end{aligned}$$

The vibration frequency f exhibits a linear relationship with the current:

$$\begin{aligned} f=k\cdot I\#\left（ 5 \right） \end{aligned}$$

Consequently, the flow rate formula can be derived as:

$$\begin{aligned} Q=\Delta V\cdot f=\pi km\left（ R^{2}-r^{2} \right）\frac{Hd\sin\theta}{R}I^{2}=C\left（ R^{2}-r^{2} \right）\frac{Hd\sin\theta}{R}I^{2}\#\left( 6 \right) \end{aligned}$$

Where $C=\pi km$ is the composite experimental coefficient, incorporating factors such as electromagnetic conversion efficiency and material elasticity.

In the formula, geometric parameters R and r directly determine the fluid channel cross-sectional area, while H, d, and θ define the spatial scope of deformation: H represents the horizontal action length, and dsinθ denotes the effective vertical deformation height. Together, these parameters influence the volume output per single deformation cycle. The current term I^2^ embodies nonlinear response characteristics: current governs both the deformation magnitude (via *α*) and the vibration frequency (via f), with their combined effect causing the flow rate to be proportional to the square of the current. This relationship is applicable in scenarios with low current, where only partial deformation occurs.

In practice, after determining geometric parameters such as R、r、H、d, and θ, different currents are applied experimentally, and corresponding flow rate data are collected. The coefficient C is then obtained through data fitting, establishing a quantitative mapping between current and flow rate. This model provides a theoretical basis for the flow control of the actuator, supporting structural optimization and control strategy design. By integrating electromagnetic actuation characteristics with geometric parameters, it enables precise quantification and regulation of fluid output.

**Supplementary Note 3** **Cultivation of plant growth explanatory notes**

The laboratory culture of wheatgrass needs to be designed based on its physiological characteristics and environmental adaptability to ensure the feasibility of the experiment and the reproducibility of the results^14^. The cultivation conditions were controlled in a constant temperature and humidity environment (temperature 25±1℃, relative humidity 60±5% RH) to avoid the interference of external environmental fluctuations on the experimental variables. The planting containers were standardized plastic pots with a diameter of 6.5 cm at the bottom and a height of 5.2 cm, and their dimensions were designed to take into account the space for root expansion and the water holding capacity of the soil, so as to ensure continuous growth from seedling stage to maturity. The soil substrate should have good air permeability and water retention, and humus is used to balance nutrient supply and drainage efficiency. The growth cycle of S. amygdalus is divided into the germination stage (about 7 days), the growth stage (8-16 days) and the maturity stage (16-22 days), with significant differences in water demand between these stages, which need to be regulated by precise irrigation strategies. A contrasting design was used, including diffuse irrigation (8 mL per day) and drip irrigation (2 Hz and 5 Hz with flow rates of 1.6 mL/min and 3.6 mL/min, respectively), which was timed to ensure uniform soil moisture and to avoid root hypoxia induced by waterlogging. High-frequency drip irrigation promotes gradient water uptake by the root system through low-flow, continuous water delivery, which significantly accelerates cell division and stem expansion during the germination and growth phases, while diffusion irrigation supports rapid longitudinal plant growth during maturity through a one-time large water supply. In the laboratory, precise control of environmental parameters eliminated external interference of temperature and humidity on growth rates, ensuring comparability between irrigation groups, and the irrigation frequency and volume were designed to match the biology of plant water use efficiency, with high-frequency drip irrigation optimizing water use during the seedling stage, and diffuse irrigation meeting the demand for high biomass accumulation during the maturity stage. The experimental design was further supported by the characteristics of wheatgrass, which has a short growth cycle that facilitates the observation of phase changes, and a high sensitivity to water that clearly reflects the effects of different irrigation strategies. In addition, the metabolic rate of the plant is stabilized at a constant temperature, which avoids the deviation of growth rate due to temperature fluctuation. Through the above standardized operation and scientific design, the experiment not only verified the advantages of drip irrigation technology in water conservation and growth promotion, but also provided a theoretical basis for large-scale cultivation and application in arid areas, reflecting the feasibility of promoting from laboratory controlled conditions to actual production scenarios^15^.

**Supplementary Note 4** **Environmental correction coefficients for water conservation and energy consumption in the Xinjiang region.**

The region's arid climate, marked by drought and low precipitation, coupled with substantial evaporation and soil salinization, underscores the challenges posed by these conditions. Conventional diffuse irrigation techniques have been shown to result in significant water loss, with as much as 50% of the water being lost to evaporation and seepage. In contrast, drip irrigation has been demonstrated to reduce this total loss to approximately 15%, a significant improvement that can be attributed to its precise water control mechanisms. To ensure accurate irrigation scheduling, it is essential to adjust the amount of water required based on the specific parameters of each irrigation method. For instance, the volume of water required for drip irrigation can be calculated as follows^11^:

$$drip irrigation water=theoretical value \times1.15$$

While the volume required for diffuse irrigation can be calculated as^11^:

$$diffuse irrigation water=theoretical value \times1.5$$

These calculations directly reflect the differences in water use efficiency between drip and diffuse irrigation techniques, highlighting the importance of adjusting the correction coefficient to account for these variations in water use efficiency within the context of Xinjiang. Cotton (Gossypium hirsutum L.)^16^ can be used as an example. Its root system is developed and drought-tolerant. A study was conducted to determine the effects of drip irrigation on cotton root growth. The experiment used a high-frequency, low-flow water supply (e.g., 5 Hz frequency of 3.6 mL/min) to simulate the natural precipitation gradient. This irrigation method was used to promote uniform water absorption by the root system and to avoid diffuse irrigation triggered by soil condensation and salt upward movement. The results showed that the practical application of drip irrigation in Shaya County cotton fields increased yields from 200-300 kg to 450 kg. This increase in yield resulted in significant water conservation. The energy consumption correction for the extreme environment in Xinjiang is determined by factors such as high temperatures and sand exacerbating the load on pumping stations. To validate the correction coefficients, the following formula is employed: total energy consumption correction for the laboratory value × 1.0625. This coefficient is introduced to account for environmental compensation and intelligent control. In terms of plant characteristics, the main crops of Xinjiang, including wheat, corn, and jujube, exhibit highly compatible growth cycles and water demand curves with the precision supply mode of drip irrigation. The unique ecological pressures and crop adaptability present in the region form the basis for the promotion of drip irrigation technology. The correction coefficients for water and energy consumption quantify the environmental constraints and provide a replicable technological paradigm for sustainable agricultural development in arid zones^17^.

**References**

1. Hashem, R. et al. Design and characterization of a bellows-driven soft pneumatic actuator. *IEEE/ASME Trans. Mechatron.* **26**, 2327-2338 (2020).
2. Qiu, Z. et al. An empirical model of soft bellows actuator. *Sci. Rep.* **14**, 28681 (2024).
3. Ministry of Water Resources of the People’s Republic of China. Annual precipitation in each province and municipality. http://www.mwr.gov.cn/sj/tjgb/szygb/202406/t20240614_1713318.html (2023).
4. Water Resources Department of Xinjiang Uygur Autonomous Region. Annual precipitation in each region. https://slt.xinjiang.gov.cn/xjslt/c114491/zfxxgk_list.shtml (2023).
5. Yang, F., Li, H. & Jiang, Y. A Review of Pressure Regulation Technologies for Irrigation Pipeline Systems. *Agriculture* **15**(14), 1528 (2025).
6. Al Nayeem, M. S., Sarker, A., Rahman, U., Roychoudhury, S. & Muthukumar, P. Energy-Efficient Automated Irrigation: A Solar-Powered Solution for Modern Agriculture. *In 2025 8th International Conference on Circuit, Power & Computing Technologies (ICCPCT)* 1915-1920 (IEEE, 2025).
7. Nalawade, S. P. & Manatkar, P. A. Smart and Water-Efficient Automatic Drip Irrigation System. *In Advanced Modelling and Innovations in Water Resources Engineering: Select Proceedings of AMIWRE 2021* 693-701 (Springer Singapore, Singapore, 2021).
8. Gallardo, M. et al. Crop growth and water use model for lettuce. *J. Irrig. Drain. Eng.* **122**, 354-359 (1996).
9. Cui, J. et al. Yield, quality and drought sensitivity of tomato to water deficit during different growth stages. *Sci. Agric.* **77**, e20180390 (2019).
10. Datta, A. et al. Water management in cotton. *Cotton Production* 47-59 (2019).
11. Zhou, H. et al. Analysis of agricultural irrigation water-using coefficient in Xinjiang arid region. Trans. *Chin. Soc. Agric. Eng.* **29**, 100-107 (2013).
12. Hou, X. et al. Determining water use and crop coefficients of drip-irrigated cotton in south Xinjiang of China under various irrigation amounts. Ind. Crops Prod. **176**, 114376 (2022)
13. Liao, Z. et al. A comprehensive thermo-viscoelastic experimental investigation of Ecoflex polymer. *Polym. Test.* **86**, 106478 (2020)
14. Steppuhn, H. et al. AC Saltlander green wheatgrass. *Can. J. Plant Sci.* **86**, 1161-1164 (2006).
15. Yin, H. et al. Soil sensors and plant wearables for smart and precision agriculture. *Adv. Mater.* **33**, 2007764 (2021).
16. Feng, L. et al. Xinjiang cotton: Achieving super-high yield through efficient utilization of light, heat, water, and fertilizer by three generations of cultivation technology systems. *Field Crops Res.* **312**, 109401 (2024).
17. Bian, Q. et al. Optimizing irrigation strategies to improve yield and water use efficiency of drip-irrigated maize in Southern Xinjiang. *Plants* **13**, 3492 (2024).
